# Translational repression of NMD targets by GIGYF2 and EIF4E2

**DOI:** 10.1371/journal.pgen.1009813

**Published:** 2021-10-19

**Authors:** Boris Zinshteyn, Niladri K. Sinha, Syed Usman Enam, Benjamin Koleske, Rachel Green

**Affiliations:** 1 Department of Molecular Biology and Genetics, Johns Hopkins University School of Medicine, Baltimore, Maryland, United States of America; 2 Howard Hughes Medical Institute, Chevy Chase, Maryland, United States of America; University of Cologne, GERMANY

## Abstract

Translation of messenger RNAs (mRNAs) with premature termination codons produces truncated proteins with potentially deleterious effects. This is prevented by nonsense-mediated mRNA decay (NMD) of these mRNAs. NMD is triggered by ribosomes terminating upstream of a splice site marked by an exon-junction complex (EJC), but also acts on many mRNAs lacking a splice junction after their termination codon. We developed a genome-wide CRISPR flow cytometry screen to identify regulators of mRNAs with premature termination codons in K562 cells. This screen recovered essentially all core NMD factors and suggested a role for EJC factors in degradation of PTCs without downstream splicing. Among the strongest hits were the translational repressors GIGYF2 and EIF4E2. GIGYF2 and EIF4E2 mediate translational repression but not mRNA decay of a subset of NMD targets and interact with NMD factors genetically and physically. Our results suggest a model wherein recognition of a stop codon as premature can lead to its translational repression through GIGYF2 and EIF4E2.

## Author summary

A premature stop codon–a signal to abort production of a protein before the normal end has been reached—is treated differently by the cell than the corresponding normal stop codon. Many disease-causing mutations result in premature stop codons, and current research suggests that differences between stop codons could be exploited for clinical therapy. We have carried out a genetic screen to identify factors that regulate the production of protein from an messenger RNA (mRNA) with a premature stop codon. Our screen recovered previously known factors in this process, as well as unexpected roles for factors that normally mark the sites of RNA splicing, as well as a pair of proteins (GIGYF2 and EIF4E2) that mediate translational repression but not mRNA decay of mRNAs with a premature stop codon.

## Introduction

Translation of messenger RNAs (mRNAs) with premature translation termination codons (PTCs) produces truncated peptides with potentially toxic dominant-negative effects [1,2]. These mRNAs are detected in a translation-dependent process called nonsense-mediated mRNA decay (NMD) [3], in which recognition of a stop codon as premature leads to degradation of the encoding mRNA [4,5]. In addition to degrading aberrant mRNAs that result from genetic mutations and mRNA processing defects, NMD regulates levels of many functional transcript isoforms [6–9] and degrades PTC-containing byproducts of regulated alternative splicing [10–12]. In cases where a PTC truncates a coding region, NMD becomes important for preventing an accumulation of truncated proteins which leads to proteotoxic stress [13].

The universal eukaryotic NMD factors (UPF1, UPF2, UPF3), which play the major role in PTC detection, were identified genetically as suppressors of frameshift mutations in *S*. *cerevisiae* [14–16], or as suppressors of multiple unrelated mutations in *C*. *elegans* [17,18]. The latter studies also identified the kinase SMG1, which phosphorylates UPF1, and additional NMD factors SMG5-7, which directly facilitate mRNA decay. The human orthologues of these factors, which have the same names, were identified by sequence homology [19–24]. Human SMG8 and SMG9 were identified biochemically as physical interactors and regulators of SMG1 [25]. More recent studies used targeted reporter assays coupled with RNAi or CRISPR to identify additional NMD factors in *C*. *elegans* and human cells [26–30], finding connections between NMD and ribosome recycling, splicing, and other processes.

In the best-studied mode of NMD, premature termination codons are distinguished from normal ones by the presence of downstream exon-junction complexes (EJCs) [31,32]. EJCs are deposited at exon-exon junctions in the nucleus during splicing, exported with the mRNA to the cytoplasm, and normally removed by translating ribosomes [33]. A stop codon sufficiently upstream of the EJC (~55 nucleotides (nt) from the exon-exon junction) prevents EJC removal and signals NMD through the interaction of EJC-associated NMD factors with NMD factors associated with the terminating ribosome [34]. EJCs effectively function as a specific (though not the only) signal for premature termination because the stop codon is located in the last exon, or within the last 55 nt of the penultimate exon, of nearly all mRNAs [32]. Some medically relevant PTCs can escape NMD when they are located in the last exon or within the last 55 nt of the penultimate exon of their transcript [35], producing truncated protein that causes dominant-negative effects. Recognition of a stop codon as premature leads to phosphorylation of the helicase UPF1 by SMG1, which in turn activates endonucleolytic cleavage by SMG6 and deadenylation/decapping through SMG5 and SMG7 [36–38].

This mechanistic model of NMD, often called the “EJC-dependent” or “EJC-enhanced” model, is complicated by the finding that depletion of NMD factors in mammalian cells stabilizes hundreds of mRNAs with no exon-exon junctions in their 3′ UTR [8], and the fact that depletion of EJC components does not always stabilize NMD reporters in *D*. *melanogaster* [22] and *C*. *elegans* [26]. These generally weaker “EJC-independent” or “3′ UTR-length dependent” targets of NMD are predicted to be recognized in part by their longer 3′ UTRs [39–41] and have been suggested to undergo inefficient translation termination and/or recycling [42,43], perhaps due to the increased distance between poly-A binding protein and the terminating ribosome [44,45]. This correlates with the functional dichotomy of NMD targets: EJC-independent “regulatory” NMD targets tend to encode full length functional protein and the cell uses the NMD pathway to adjust their levels, whereas “degradative” NMD transcripts with 3′ UTR introns tend to encode nonfunctional truncated proteins, which must be repressed as much as possible in order to minimize proteotoxic stress [46]. The EJC-independent pathway appears to be the only form in budding yeast, which exhibit robust NMD facilitated by the core factors UPF1-3, despite having few spliced mRNAs and no known EJC components [47]. For EJC-independent NMD, it has been proposed that recognition of NMD targets depends on accumulation of some factor, possibly UPF1, in the 3′ UTR [48–50]. This model is supported by the fact that small amounts of stop codon readthrough dramatically reduce degradation of an mRNA by NMD by repeatedly clearing the 3’UTR of RNA-binding proteins [30,50–52].

Translational repression is another crucial mechanism for regulation and quality control of cellular mRNAs. Translational repression often precedes or accompanies the mRNA decay and/or proteasomal degradation mediated by numerous mRNA regulatory features, including miRNA target sites, AU-rich elements, and ribosome stalling sequences [53–58]. Repression of NMD targets at the translational level has been proposed by multiple groups [59,60] and even hypothesized to be a necessary pre-requisite for decapping and degradation of the mRNA. However, evidence for a specific pathway mediating translational repression of NMD targets is lacking.

Previous reporter screens have looked for the regulators of natural intron sequences that trigger EJC-dependent NMD. We sought to gain more general insights into PTC recognition and regulation by studying the case of a PTC without downstream 3′ UTR splicing. We anticipated the identification of RNA-binding proteins and translation factors that establish or detect the protein contexts for this mode of NMD. To screen for these factors, we developed a fluorescent reporter to perform a whole-genome CRISPR screen. This screen identified many of the known NMD factors and surprisingly identified a role for core EJC subunits in regulation of NMD targets even in the absence of 3′ UTR splicing. Our screen also identified the translational repression complex GIGYF2•EIF4E2 as a strong regulator of our reporter. Ribosome profiling revealed that a subset of NMD targets is translationally repressed by these factors and chemical epistasis experiments suggest that this repression requires the NMD machinery. Therefore, GIGYF2•EIF4E2 adds a failsafe layer of regulation for NMD-targeted transcripts to protect against the production of truncated proteins and subsequent proteotoxic stress.

## Results

### A reporter for NMD independent of 3′ UTR splicing

**Fig 1:**
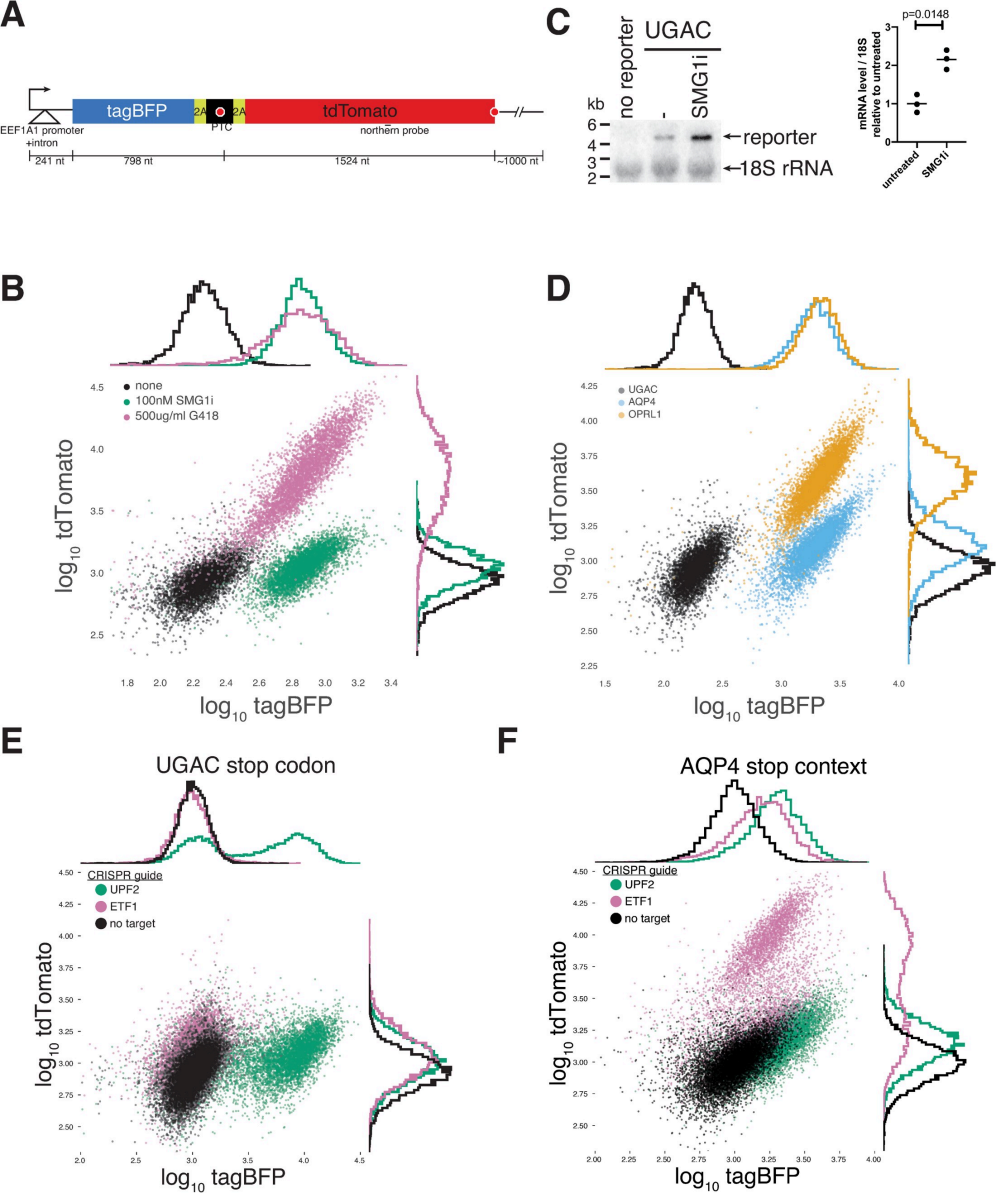
A fluorescent reporter for nonsense-mediated mRNA decay and stop codon readthrough. A) Dual fluorescence reporter used in this study. 5′ UTR length is based on the annotated start site of the human EEF1A1 transcript. 3′ UTR length is estimated from nanopore direct RNA sequencing data (S2D Fig). Position of northern probe for panel C is indicated. B) Flow cytometry of monoclonal K562 cells expressing reporter with UGAC PTC, treated with 500 μg/ml G418 or 300 nM SMG1i for 24 hours. C) (left) Northern blot of parental K562 cell line, and UGAC reporter line untreated or treated with 300 nM SMG1i for 4 hours, using probe oBZ732 against the tdTomato coding region (panel A). The off-target lower band comigrates with the 18S rRNA. (right) Quantification of reporter band from triplicate blots, relative to 18S band in the same lane. The mean of three WT replicates was set to 1. P values were computed by two-tailed ratio paired t-test. D) Flow cytometry of monoclonal K562 cells expressing reporter with different stop codon contexts cloned into the PTC location. The AQP4 and OPRL1 contexts are expected to cause 5% and 15% readthrough, respectively. E) Flow cytometry of UGAC PTC reporter line lentivirally transduced with CRISPR guides targeting the coding region of the indicated genes. F) Same as panel E for AQP4 PTC reporter.

To screen for factors involved in 3′ UTR-length dependent NMD, we created a dual-fluorescence reporter consisting of a tagBFP coding region, fused in-frame to the coding region of tdTomato (Fig 1A). When a stop codon is inserted between these two coding regions, the reporter models a transcript with a coding region interrupted by a PTC, with the tdTomato sequence serving as a long 3′ UTR to trigger NMD. There is only one intron in the reporter, upstream of the start codon, so we anticipated that no EJCs would be deposited downstream of the PTC. The PTC site is flanked by foot and mouth disease virus 2A peptide-bond skipping sequences [61–64]. These trigger skipping events that separate the tagBFP signal from the possible effects of protein quality control or differences in protein stability or function caused by the sequences at the PTC [65–68]. The tagBFP output from the reporter depends on the overall mRNA level of the reporter as well as its translation, while the tdTomato signal monitors stop codon readthrough. Preliminary experiments showed that tagBFP and tdTomato are well-separated by flow cytometry (S1A Fig), and the 2A peptides function efficiently, predominantly producing monomeric tagBFP (S1B Fig). For our baseline PTC sequence, we chose UGAC, which is the most likely of the stop tetranucleotides to allow stop codon readthrough and is enriched in NMD targets [52], but is still commonly used at normal human termination codons [69,70]. We lentivirally integrated this reporter into nonadherent K562 (human lymphoblastoid leukemia) cells and isolated a monoclonal line expressing the reporter. To confirm that the reporter mRNA was an NMD target we used the small-molecule NMD inhibitor SMG1i, which prevents phosphorylation of UPF1 by SMG1 [71]. Treating the reporter cells with SMG1i for 24 hours increased their median tagBFP signal four-fold, indicating that the reporter was indeed an NMD target (Fig 1B). A shorter 4-hour treatment with SMG1i increased the reporter mRNA level by 2-fold (Fig 1C), further confirming that it is an NMD target. This shorter treatment was possible because we did not need to wait for fluorescent protein maturation to measure mRNA levels. Incubation of these reporter cells with G418 led to a 6.8-fold increase in median tdTomato output from the reporter, as well as a concomitant 3.8-fold increase in median tagBFP fluorescence, consistent with the model that robust readthrough of the PTC of this reporter suppresses its NMD (Fig 1B).

To further explore the effects of stop codon readthrough on mRNA levels of this reporter, we cloned 3 additional sequences into the PTC location: a UGG tryptophan codon, and the stop codon contexts of the human AQP4 and OPRL1 genes, which were previously shown to confer 5 and 15% readthrough, respectively [67,72]. Although these monoclonal lines are not directly comparable due to likely differences in integration, both tagBFP and tdTomato levels increased with the expected increase in stop codon readthrough (Figs 1D and S2A). The fact that tagBFP levels increased more than tdTomato levels is consistent with previous results showing that small amounts of stop codon readthrough are sufficient to substantially suppress NMD [50]. As expected from their high rates of basal stop codon readthrough, these leaky stop codon contexts showed little or no additional readthrough stimulation by G418 or NMD suppression by SMG1i, particularly in the case of OPRL1 (S2B and S2C Fig).

To assess the suitability of these lines for genome-wide screening by CRISPR-Cas9 [73–75], we lentivirally integrated *Streptococcus pyogenes* Cas9 into these cells, followed by guide RNAs against UPF2 (essential for NMD) or ETF1 (encoding eRF1, the catalytic eukaryotic termination factor). We performed flow cytometry 5 days after infection, which included 2 days of puromycin selection to enrich for cells expressing the guides. Depletion of UPF2 in the UGAC reporter line increased tagBFP activity 7.6-fold (Fig 1E) consistent with UPF2’s key role in NMD. UPF2 depletion led to a smaller increase in BFP levels from the AQP4 reporter (Fig 1F), likely due to the already reduced activity of NMD for this reporter. The bimodal distributions of fluorescence intensities observed in these CRISPR experiments was likely due to a subset of cells that did not undergo editing, or in which editing caused mutations that do not affect gene function. Depletion of eRF1 did not substantially increase tdTomato levels for UGAC, suggesting that near-cognate tRNAs do not readily access this stop codon, even with reduced levels of competing release factor. On the other hand, the AQP4 reporter showed a 5.3-fold increase in tdTomato fluorescence upon eRF1 depletion, indicating that this less efficient stop codon is sensitive to release factor levels. Based on these results, we decided that the UGAC reporter would provide a suitable background to screen for NMD suppression.

### A PTC reporter screen recovers core NMD factors, and additional regulators

**Fig 2:**
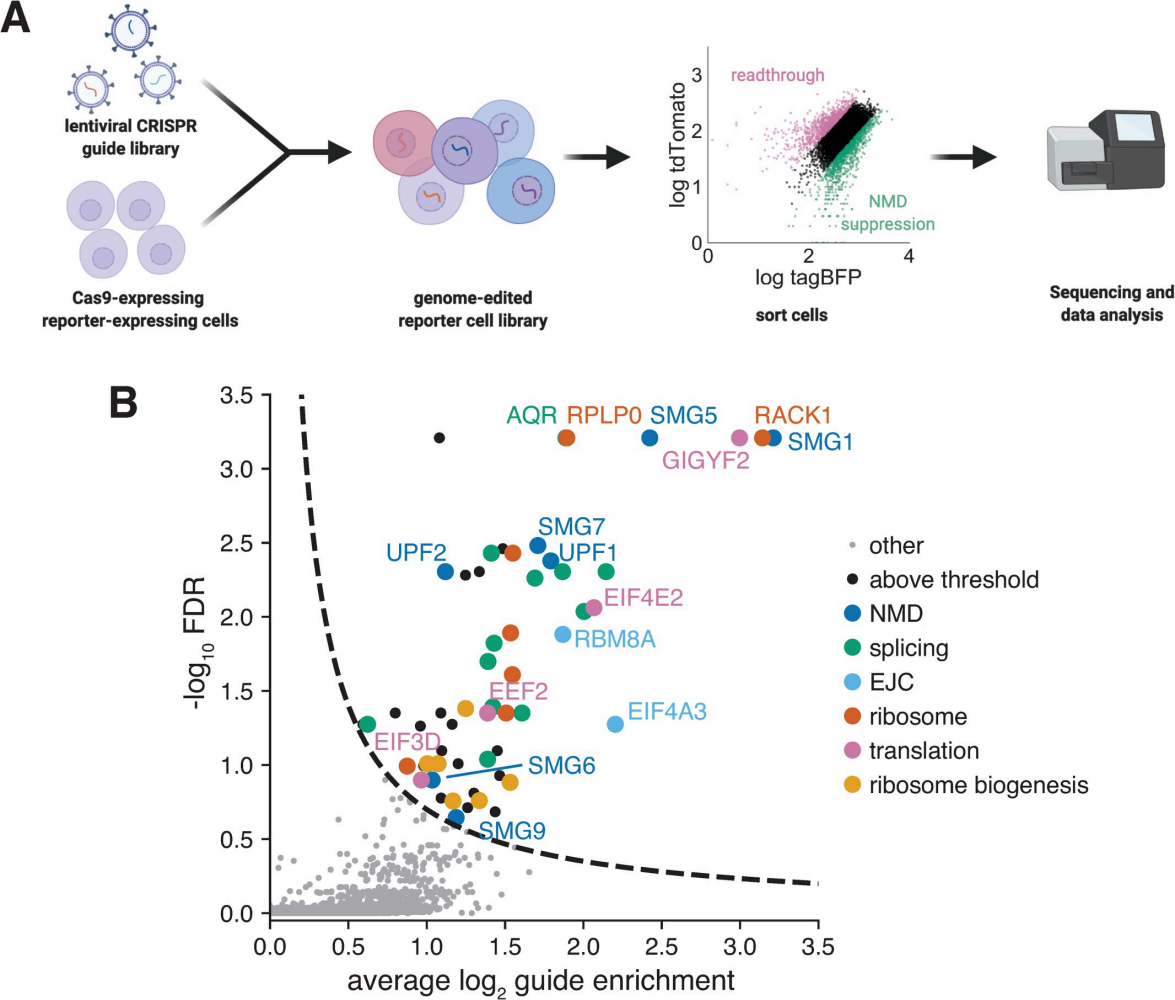
A screen for genes that repress a PTC-containing mRNA. A) Flowchart of genome-wide CRISPR screening of fluorescent reporter by FACS. B) Volcano plot of fold enrichment and false discovery rate (FDR) for CRISPR guides sequenced from cells with decreased tdTomato/tagBFP ratio compared to the input population for the UGAC reporter. Hits were manually annotated into the indicated categories. Values computed with MAGeCK [76].

To identify genes involved in 3′ UTR length dependent NMD, we performed genome-wide CRISPR flow cytometry screens. We transduced our UGAC and AQP4 reporter lines with the Brunello lentiviral library of 77,441 guide RNAs targeting 19,114 human genes [74,75], selected for guide integration using puromycin, and used fluorescence-activated cell sorting (FACS) to isolate the bottom 10% of cells based on their tdTomato/tagBFP ratio 6 days after transduction (Fig 2A). Based on our control experiments, NMD suppression decreased this ratio, while stop codon readthrough increased it (S2D Fig). We sequenced the CRISPR guides from these isolated cells and compared their prevalence between the sorted and unsorted population using MAGeCK [76].

The screen performed with the normal UGAC stop codon (Fig 2B and S1 Table) enriched most of the core NMD machinery, including UPF1, UPF2, SMG1, SMG5, SMG6, SMG7 and SMG9. The AQP4 screen (S3A Fig and S2 Table) identified fewer significant hits, possibly due to this reporter’s reduced dynamic range, but these hits still included the NMD factors UPF1, UPF2, UPF3B, SMG1, SMG5, SMG6 and SMG9. Identification of the canonical NMD factors further confirms that our reporters are NMD targets and that the screen is working as expected with high sensitivity. We performed a preliminary validation of 23 hits from the NMD screen. To do so we recloned the 2 most-enriched CRISPR guides from our screen for each of these genes, individually integrated them into our UGAC reporter line and performed flow cytometry after puromycin selection. We repeated these infections multiple times for each guide and compared the mean tagBFP intensity of the resulting cells to those infected with 2 separate nontargeting guides to establish statistical significance (S3B Fig and S3 Table). Of the tested genes, 17 had a significant (adjusted p<0.05) increase in tagBFP signal, indicating that many of the screen hits are likely to have real effects on reporter signal.

### Some transcripts without 3′ UTR introns are derepressed when EJC components are depleted

In addition to the core NMD factors, a number of splicing factors, as well as two core components of the exon-junction complex (RBM8A/Y14 and EIF4A3), are enriched in both screens (Figs 2B and S3A and S3B) despite the intentional omission of splice sites downstream of the premature stop codon. It is likely that the other core EJC factor MAGOH was not detected because it is encoded by two redundant genes [77]. To test the PTC dependence of these hits, we repeated some of the infections in 3 different reporter lines: the UGAC reporter, AQP4 reporter, and the UGGC reporter which has no PTC (S3C Fig). These sgRNAs cause the largest increase in tagBFP with the UGAC reporter, a weaker effect with the AQP4 stop context and no effect without a PTC, indicating that the effect of knocking out these genes depends on a PTC.

To rule out the existence of unexpected splice sites (and the corresponding unexpected EJCs) in our reporter, we performed nanopore direct RNA sequencing on HEK293FT cells transfected with the UGAC or AQP4 reporter plasmids (S3D Fig). We were unable to detect unexpected splice products downstream of the PTC, as evidenced by a lack of detectable gapped reads outside of the expected 5′ UTR intron (black arcs in S3D Fig). We were similarly unable to detect aberrant splicing by short-read RNA-seq upon treatment of the UGAC reporter cell line treated with SMG1i, which should stabilize any aberrant splice isoforms targeted by NMD (S3E Fig). An alternative explanation for these results is that the effect of these EJC factors on our reporter is the result of EJC components placed independently of splicing. While CLIP peaks for EJC components have been detected in non-canonical positions [78–80], their biological relevance remains uncertain. To further explore this possibility, we analyzed RNA-seq data from the ENCODE consortium [81], in which the EJC factors EIF4A3 or MAGOH had been knocked down with RNAi in HEPG2 or K562 cells. We determined a set of NMD target transcripts from the data of [82], which we divided into EJC-dependent (with a 3′ UTR intron more than 50 nt downstream of the stop codon), and EJC-independent targets. We further divided the EJC-independent group into those with or without an upstream open reading frame, which could trigger EJC-dependent NMD. In this regard, we chose to be overly broad, defining any mRNA with an out-of-frame AUG codon in its 5′ UTR as containing a uORF. As expected, knockdown of either EJC factor increased the mRNA levels of NMD targets with 3′ UTR introns compared to other transcripts in both cell lines (S4A Fig). However, we also observed an increase in the mRNA levels of NMD targets without 3′ UTR introns, regardless of the presence of a uORF (S4A Fig). Moreover, we observed increases in the mRNA levels of several NMD factors which were previously proposed to be autoregulated by EJC-independent NMD (S4B and S4C Fig) [8]. These findings differ from a previous study in HeLa cells which found that only UPF1 and SMG5 increased upon eIF4A3 knockdown [83] and concluded that some of these targets were EJC-dependent and others were EJC-independent. Together, these results suggest that EJC components also regulate the levels of mRNAs that do not obviously follow the 50 nt rule [32].

### GIGYF2•EIF4E2 represses NMD reporter output

Our screen identified several core translation factors and ribosomal proteins which increased tagBFP production from our reporter when depleted (Figs 2B and S3A–S3C). The presence of these factors reflects the well-described requirement for active translation for NMD. However, among the most highly enriched genes were two factors that comprise a selective translational repression complex with no known connection to NMD: GIGYF2 (GRB10 interacting GYF protein 2) and EIF4E2 (eukaryotic initiation factor 4E2, also known as 4E homologous protein, or 4E-HP) [84], as well as the small ribosomal subunit protein RACK1. We were intrigued by the presence of RACK1, GIGYF2 and EIF4E2 in our screen results as they have been implicated in the cellular response to ribosome stalling and collisions but not NMD, so we focused on these factors in our further characterization.

**Fig 3:**
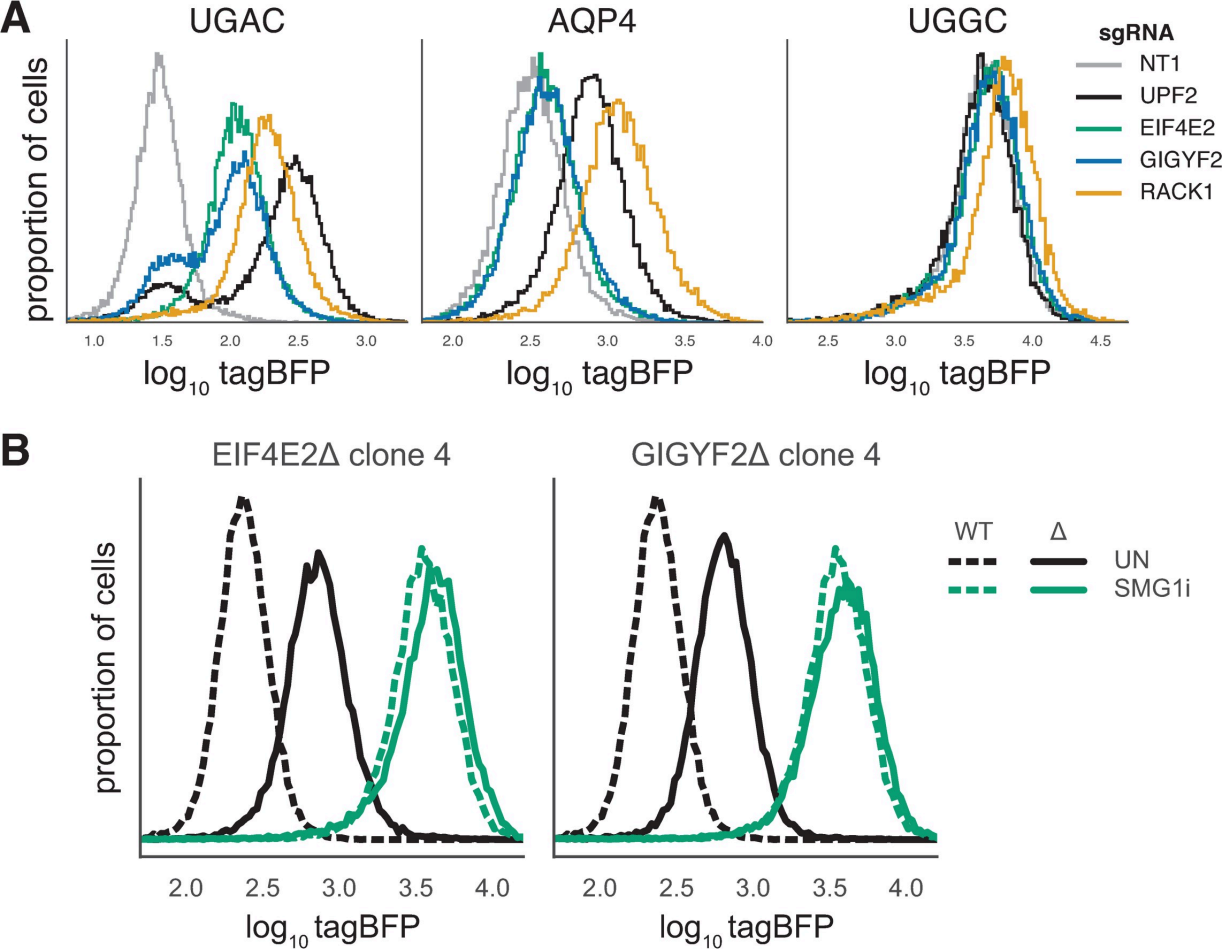
GIGYF2 and EIF4E2 do not affect reporter expression in the presence of SMG1i. A) Histograms of tagBFP intensity from flow cytometry of reporter lines containing UGAC stop codon, AQP4 stop context and no stop codon at the PTC position, electroporated with a plasmid expressing nontargeting CRISPR guide (NT1) or guides against the indicated genes. Flow cytometry was performed 5 days after electroporation. B) Representative distributions of tagBFP from flow cytometry of UGAC reporter in WT (black dotted line) background and EIF4E2Δ/GIGYF2Δ monoclonal lines (black solid line), as well as the same lines treated with SMG1i (green lines).

EIF4E2 is a homolog of the canonical cap-binding protein EIF4E that can bind the m^7^G cap but cannot interact with the translation initiation scaffold protein EIF4G, disconnecting the cap from the rest of the translational machinery [85–87]. This leads to reduced translation initiation and/or increased mRNA decay of the mRNAs to which EIF4E2 is recruited. GIGYF2 recruits itself and EIF4E2 to specific mRNAs by interacting with RNA-binding proteins containing proline-rich sequence motifs [88]. We validated these strong candidates by electroporation of sgRNA plasmids targeting RACK1, GIGYF2 and EIF4E2 into the UGAC reporter line, which caused a strong increase in tagBFP signal (Fig 3A). The AQP4 and no-PTC (UGGC) lines respectively responded weakly and not at all to GIGYF2 or EIF4E2 depletion, while RACK1 depletion caused a small increase even with the no-PTC (UGGC) reporter. These observations indicate that regulation of the reporter by RACK1 is at least in part PTC-independent, while the GIGYF2•EIF4E2 repression is strongly dependent on the presence of a premature stop codon. Moreover, the generally weaker response of the reporter to GIGYF2 or EIF4E2 knockout compared to UPF2 indicates that the former two factors are not central to NMD of this reporter.

### Previously identified RQC factors ZNF598 and EDF1 are not required to repress the PTC reporter

RACK1 is important for the ubiquitination and rescue of ribosomes as well as degradation of nascent peptides by ribosome-associated quality control (RQC) on extreme ribosome stalls [89–92] and is located at the interface of collided ribosomes [93,94]. GIGYF2•EIF4E2 has also been implicated in RQC, wherein it is recruited by EDF1 to sites of ribosome collision [55,95] to prevent further translation of the mRNA. GIGYF2 and EIF4E2 have also been extensively studied as translational repressors in other contexts. EIF4E2 is recruited by tristetraprolin (TTP) to mRNAs with AU-rich elements in their 3′ UTR [53,56,58] and by AGO2 to miRNA targets [96,97]. Because of the previously described roles of both RACK1 and GIGYF2•EIF4E2 in RQC, we tested if EDF1 was required for repression of our reporter. Depletion of EDF1 had a modest effect on reporter expression relative to depletion of GIGYF2 or EIF4E2 (S5A Fig). These observations suggest that the role of GIGYF2•EIF4E2 on our PTC-containing reporter is distinct from its recently described role in RQC which is completely dependent on EDF1 [55,95]. We further considered the possibility that GIGYF2•EIF4E2 is recruited to our reporter by ZNF598, the E3 ubiquitin ligase that is essential for RQC and has also been reported to interact with and recruit these factors to some mRNAs [58,84,98]. Targeting ZNF598 with sgRNAs had no effect on our reporter’s expression (S5B Fig), despite robust reduction of ZNF598 protein levels (S5C Fig).

Since 2A peptides were previously shown to cause ribosome stalling [64], we tested the possibility that the effects of GIGYF2•EIF4E2 on our reporter were the result of ribosome collisions at the 2A peptide sequence upstream of the PTC. We generated a new K562 monoclonal reporter line with a 2A peptide mutation that was previously shown to abrogate 2A function and ribosome stalling at the 2A sequence [64]. We integrated Cas9, electroporated these lines with sgRNA plasmids, and repeated the flow cytometry assay. We found that individual depletions of UPF2, GIGYF2, EIF4E2 and RACK1 all still increased tagBFP signal from this reporter (S5D Fig), indicating that the action of these genes on this reporter is not solely the result of ribosome stalling at the 2A peptide sequence. The fact that EDF1 depletion had little effect on the 2A-less reporter suggests that the modest effect previously observed was the result of ribosome collisions on the 2A peptide, but the substantial residual effect of GIGYF2 or EIF4E2 depletion is independent of these collisions.

### GIGYF2•EIF4E2-mediated repression of a PTC-containing mRNA depends on functional NMD machinery

Having established the dependence of GIGYF2•EIF4E2 function on a premature termination codon, we next wished to determine the mechanism of reporter repression by GIGYF2•EIF4E2 and to explore its interaction with the NMD pathway. We generated stable knockout lines for these factors and isolated the population of cells with increased tagBFP levels by FACS from our GIGYF2, EIF4E2, RACK1 and UPF2 guide infections. We were easily able to generate polyclonal GIGYF2Δ and EIF4E2Δ knockout lines whose increased tagBFP levels were stable over weeks, indicating a minimal effect on cell growth or viability. By contrast, our RACK1Δ and UPF2Δ polyclonal lines generally reverted to wild-type levels of tagBFP over time (S6A Fig), consistent with the importance of NMD factors and ribosomal proteins for cell growth and viability. From these polyclonal lines, we generated GIGYF2Δ and EIF4E2Δ monoclonal lines that we use for subsequent experiments (S6B Fig). Sequencing of the sgRNA target sites in these monoclonal lines revealed frameshift mutations in all alleles (S6C Fig).

It is possible that the increase in tagBFP levels of our reporter upon deletion of GIGYF2 or EIF4E2 was the result of a global increase in translation in the cell. To test this possibility, we fractionated polysomes from WT cells as well as our GIGYF2Δ and EIF4E2Δ polyclonal lines on 10–50% sucrose gradients and measured the ratio of polysomes to free subunits, a measure of bulk translational activity. We found no difference between the knockout lines and our WT reporter line (S6D Fig), indicating that GIGYF2•EIF4E2 is not globally repressing translation of cellular mRNA in our cell lines.

Since regulation of our reporter by GIGYF2•EIF4E2 depends on the presence of a PTC, we tested whether it also depends on the activity of the NMD machinery. Genetic epistasis experiments were not possible due to the essentiality of the core NMD factors in human cells. Instead, we treated WT, EIF4E2Δ and GIGYF2Δ reporter cell lines with SMG1i to inhibit phosphorylation of UPF1 (and thereby block its NMD function) in these lines. If UPF1 phosphorylation is required for the activity of GIGYF2•EIF4E2 on this reporter, then knockout of EIF4E2 or GIGYF2 genes should have no additional effect in SMG1i-treated cells. In the untreated condition, the GIGYF2Δ/EIF4E2Δ polyclonal and monoclonal lines showed similar ~2.5-fold increases in median tagBFP fluorescence compared to the WT line (Fig 3B). SMG1i treatment increased tagBFP fluorescence (~15 fold for the WT line) in both WT and knockout cell lines, indicating that GIGYF2•EIF4E2 is not required for SMG1-mediated repression of this reporter (Figs 3B and S6E). SMG1i treatment eliminated the difference between the WT and mutant lines (Figs 3B and S6E and S6F), consistent with a model where phosphorylation of UPF1 is required for GIGYF2•EIF4E2-mediated repression of the reporter.

### GIGYF2•EIF4E2 translationally represses a subset of endogenous NMD targets

To test if our reporter was regulated at the level of translation or mRNA abundance, and to globally examine the effects of GIGYF2•EIF4E2 on NMD targets and other mRNAs, we performed matched ribosome footprint profiling and RNA-seq on WT, GIGYF2Δ and EIF4E2Δ monoclonal lines, as well as these same lines treated with SMG1i for 4 hours. Two biological replicates were performed for each sample, and we obtained approximately 2x10^7^ and 4x10^6^ transcript-mapping reads per replicate for RNA-seq and ribosome profiling, respectively (S4–S13 Tables).

**Fig 4:**
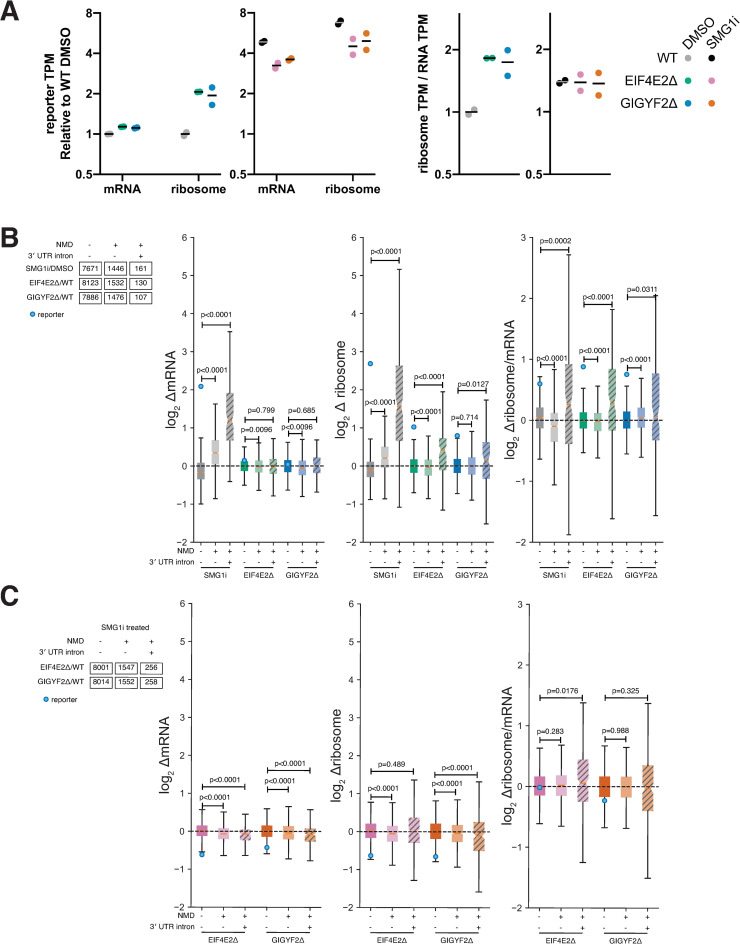
Translational repression of NMD targets by GIGYF2 and EIF4E2. A) Left: Transcripts per million (TPM) for reporter computed from RNA-seq and ribosome profiling data, normalized to the mean of DMSO-treated WT cells, for WT, EIF4E2Δ monoclonal and GIGYF2Δ monoclonal reporter lines treated with 300nM SMG1i or an equal volume of DMSO for 4 hours. Right: Normalized ratio of ribosome profiling to RNA-seq TPMs for same samples. B) Fold changes in mRNA levels, ribosome footprints and ribosome occupancy (ratio of ribosome/mRNA) in SMG1i treated cells (gray), EIF4E2Δ (green) or GIGYF2Δ (blue) cells, relative to WT cells treated with DMSO, for all transcripts (dark bars), empirically determined NMD targets (faded bars), and the subset of these targets with 3′ UTR introns (faded hatched bars). Transcript counts for each set are indicated at left, and p values from Mann-Whitney U-test are indicated. Box plots show the inter-quartile range, a red line indicates the median. Notches show the 95% confidence interval of the median. Whiskers show the data range, up to 1.5 times the interquartile range. C) As in panel B, except SMG1i-treated mutants are compared to SMG1i-treated WT cells. EIF4E2Δ is in pink and GIGYF2Δ in vermilion.

Each of the profiled cell lines expressed the UGAC reporter, allowing us to assess its translation in these conditions. SMG1i treatment increased reporter mRNA levels 4.9-fold but increased ribosome footprints 6.8-fold (Fig 4A). This 40% increase in ribosome occupancy (ratio of normalized ribosome footprint reads to mRNA reads) indicates that the reporter undergoes translational repression that depends on the kinase activity of SMG1. EIF4E2 or GIGYF2 knockout increased reporter mRNA levels by only 13% but increased ribosome footprints by 2-fold (Fig 4A), indicating that these proteins primarily affect translation of the reporter and are not critical for NMD. GIGYF2 and EIF4E2 knockout did not increase translation of the reporter relative to WT in the presence of SMG1i (Fig 4A). These observations from sequencing approaches agree with the fluorescence signals from the reporter in the same cell lines (Fig 3), further confirming that translational repression of this reporter by GIGYF2•EIF4E2 depends on phosphorylation of UPF1.

We next investigated the effect of these same depletions and treatments on known NMD targets. Unlike our reporter, we expected most of the strongest natural NMD targets to be alternative splice isoforms, which are difficult to quantify with ribosome profiling. To improve our ability to assign ribosome fragments to alternative transcript isoforms, we used ORFquant [99] to generate a limited set of transcript annotations with evidence of translation, which we further filtered to choose one isoform per stop codon position. We used previously-published RNA-seq data from knockdowns of several NMD effector proteins in HeLa cells [82] to define NMD-targeted transcripts with and without 3′ UTR introns (our working definition for EJC-dependent and EJC-independent, respectively) within this set of transcripts. We used Salmon [100] to estimate RNA-seq and ribosome footprint read counts for transcripts and coding regions, respectively (S5 and S6 Tables). Finally, we used the Xtail package [101] to compute ribosome occupancy fold changes and statistical significance (S7–S13 Tables).

As expected, treatment with SMG1i caused increased mRNA levels and ribosome-protected mRNA fragments for NMD target transcripts, with a larger increase for transcripts with 3′ UTR introns (Fig 4B). SMG1i treatment also increased the ribosome occupancy (ratio of ribosome footprints to mRNA) of the subset of NMD targets with 3′ UTR introns. Knockout of neither GIGYF2 nor EIF4E2 increased the mRNA levels of either class of NMD targets but both knockouts increased the ribosome footprints and average ribosome occupancy for NMD targets with 3′ UTR introns compared to transcripts that are not NMD targets (Fig 4B). While there were some statistically significant changes for NMD targets without 3′ UTR introns, the magnitude of these changes was small and not consistent in direction between GIGYF2Δ and EIF4E2Δ lines. We observed a similar increase in ribosome occupancy on the group of NMD targets with 3′ UTR introns in EIF4E2Δ or GIGYF1Δ/GIGYF2Δ HEK293T cells in data acquired by Weber *et al*. [57] (S7A Fig). No such affect was observed in the ZNF598Δ cells profiled in the same study (S7A Fig), consistent with the lack of effect of ZNF598 knockout on our PTC reporter line (S5B Fig).

To see if the observed translational repression of select NMD target transcripts by GIGYF2•EIF4E2 was dependent on the NMD machinery, we compared the ribosome profiling data from our SMG1i-treated GIGYF2Δ and EIF4E2Δ cells to the SMG1i-treated WT cells (Fig 4C). In this comparison NMD targets showed little or no increase in ribosome occupancy, similar to the effects on our reporter. These results agree with the fluorescence and ribosome profiling data for our reporter and suggest that the observed repression of these transcripts via GIGYF2•EIF4E2 depends on the NMD machinery.

**Fig 5:**
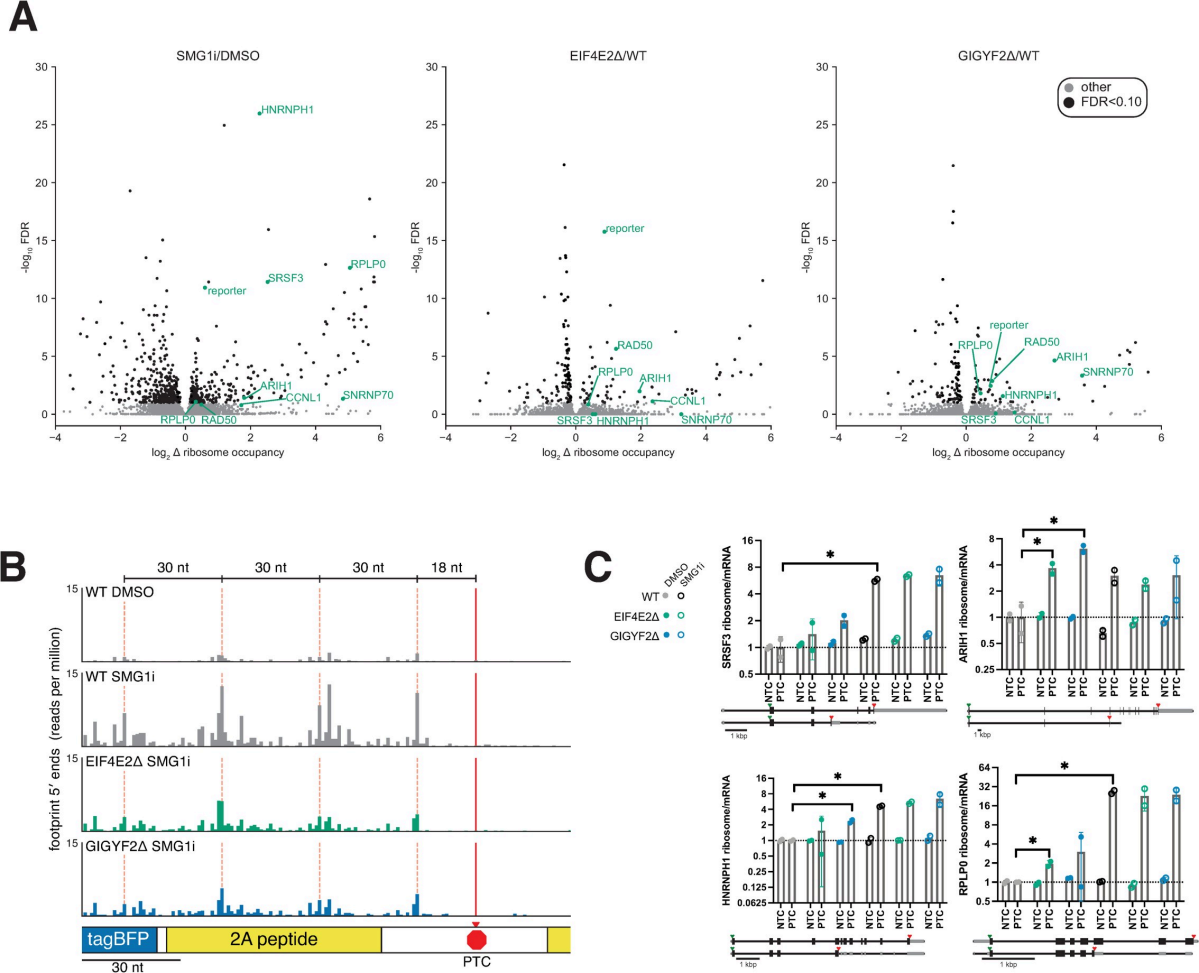
Targets of translational repression in GIGYF2Δ and EIF4E2Δ. A) Volcano plot comparing log_2_ ribosome occupancy fold changes and -log_10_ FDR, as computed by Xtail, for transcripts in the indicated knockouts or treatment. Transcripts passing an FDR cutoff of 0.1 are in black. Transcripts mentioned in the text are highlighted in green, with gene name indicated. B) Distribution of 5′ ends of ribosome footprints upstream of the PTC in our UGAC reporter. There is an 18nt offset from the last nucleotide of the coding region (red arrow) and the 5′ end of ribosome footprints on the stop codon. C) Quantification of the ratio of ribosome/mRNA TPMs for PTC and NTC transcripts of example target genes identified in Fig 5A. Two-tailed t-test p values of <0.05 are indicated by *.

We identified specific transcripts with differences in ribosome occupancy in GIGYF2Δ and EIF4E2Δ for more detailed analysis. We were able to identify 107 transcripts with significant ribosome occupancy increases in GIGYF2Δ or EIF4E2Δ (Figs 5A and S7B). The 23–25% overlap between the GIGYF2Δ and EIF4E2Δ transcript sets (S6C Fig) is comparable to the 16–24% overlap observed for mRNA level changes between similar knockouts in HEK293T cells [57], and is unsurprising considering both the increased uncertainty of determining changes in ribosome occupancy compared to changes in mRNA levels, and the fact that these factors are members of multiple protein complexes with nonoverlapping functions [56,58,84,96,97,102]. 16 of the transcripts with increased ribosome occupancy in GIGYF2Δ or EIF4E2Δ also had increased ribosome occupancy in SMG1i treatment–an overlap larger than would be expected strictly by chance (S6C Fig). We also found that EJC-dependent NMD targets had higher than average ribosome occupancy (S7D Fig), even more so than our identified translational targets of EIF4E2 and GIGYF2 (S7E Fig) suggesting that increased ribosome occupancy could be a trigger for this regulation, consistent with previously-defined roles for GIGYF2•EIF4E2 in responding to ribosome collisions on crowded mRNAs [55,57,95]. Further, we observed a 30 nt periodicity of ribosome footprints starting 18 nts upstream of the PTC on our reporter mRNA, consistent with ribosome collisions at this stop codon (Fig 5B), and a clear indication that multiple ribosomes occupy this NMD target mRNA. This result is also consistent with single-molecule data showing that multiple termination events are generally required to trigger NMD on a reporter mRNA [103], which was typically occupied by multiple ribosomes. We were unable to examine ribosome positions upstream of other PTCs due to the complexities of assigning ribosome footprints to alternative rare splice isoforms. Other NMD transcripts affected by GIGYF2•EIF4E2 included PTC-containing isoforms of several endogenous genes (RAD50, CCNL1, ARIH1, HNRNPH1, SRSF3, RPLP0, SNRNP70). Splice isoforms of SRSF3, ARIH1, HNRNPH1 and RPLP0 that encode a PTC had increased ribosome occupancy upon knockout of GIGYF2 or EIF4E2, as well as during SMG1i treatment (Fig 5C). The ribosome occupancy on isoforms of the same genes without a PTC was not affected. As before, there was little or no further effect of the GIGYF2 or EIF4E2 knockout on ribosome occupancy in the SMG1i-treated conditions. SRSF3 is particularly notable, since it is a member of a well-described class of SR-protein transcripts that encode an ultraconserved “poison cassette exon,” which contains a PTC that targets the transcript for NMD. Inclusion of the exon is autoregulated by the SR splicing regulator encoded by the transcript [10–12]. This provides an elegant mechanism by which these proteins use alternative splicing and NMD to regulate their own levels. Our results suggest that this regulatory mechanism includes translational repression in addition to NMD to reduce production of the nonfunctional truncated SR protein.

### GIGYF2•EIF4E2 physically interacts with UPF1 in an RNA-dependent fashion

**Fig 6:**
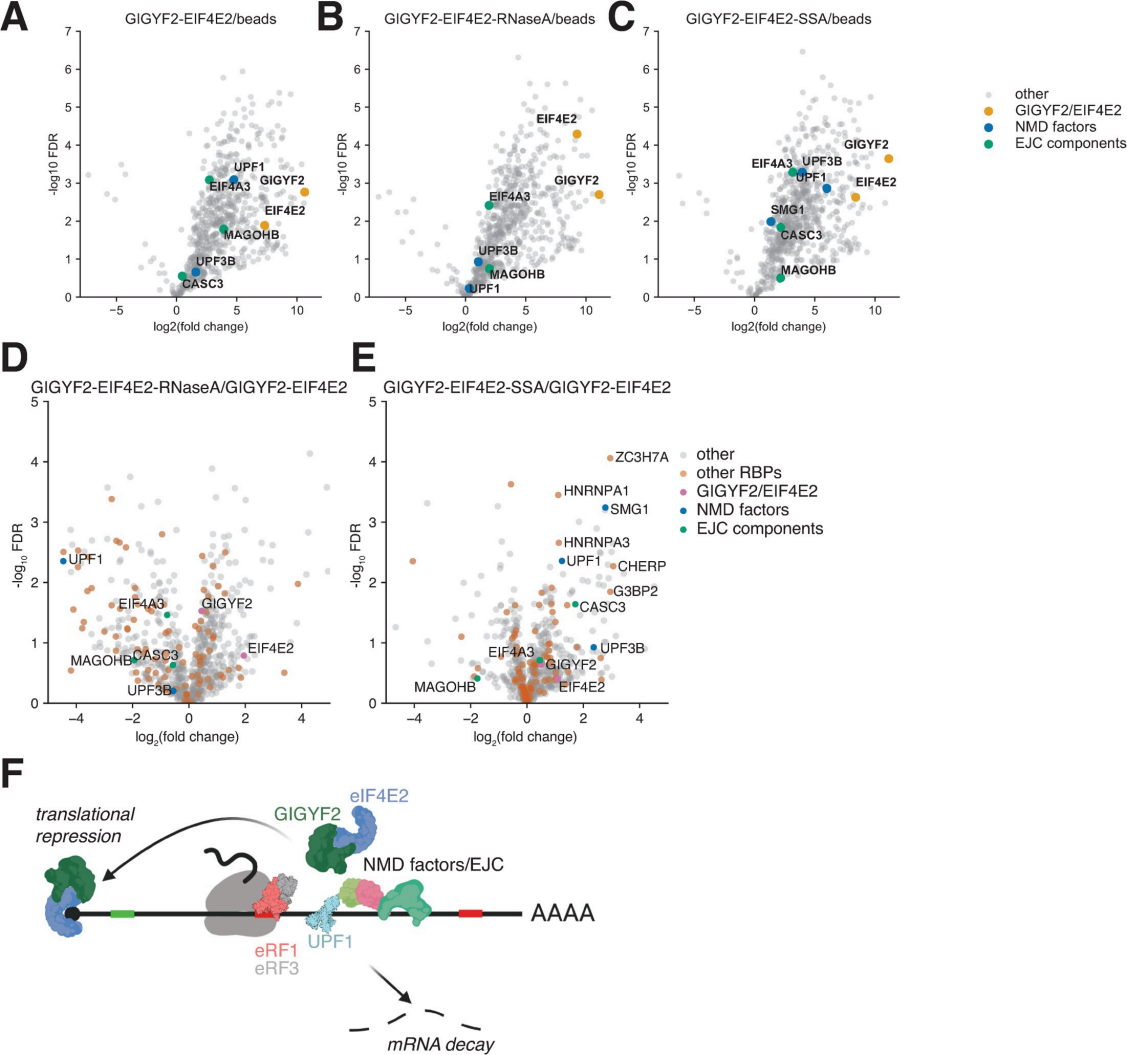
Physical interaction between UPF1 and GIGYF2•EIF4E2. A) Interaction analyses of StrepII-GIGYF2. Volcano plot showing change and p value of LFQ intensity of proteins in StrepII-GIGYF2 IP compared to a mock transfection in HEK293T cells. B) Same as panel A, with RNase A treatment of lysates or C) Treatment of cells with Spliceostatin A before harvest. D) Comparison of RNase treated GIGYF2 IP with untreated. List of highlighted RBPs downloaded from the RNA binding protein database on 7/17/2021 [125]. E) Comparison of spliceostatin A treated GIGYF2 IP with untreated. F) Model for translational repression of NMD targets by GIGYF2•EIF4E2.

The chemical genetic interaction between GIGYF2•EIF4E2 and SMG1i on our reporter and on endogenous NMD targets raised the possibility of a physical interaction between GIGYF2•EIF4E2 and the NMD machinery. We found evidence for an interaction between GIGYF2•EIF4E2 and UPF1 in previously-published UPF1 immunoprecipitation (IP) mass spectrometry data from HEK293T cells [104], in a GIGYF2 IP in HeLa cells [105], and an EIF4E2 proximity-dependent Biotin identification (BioID) experiment in HEK293 cells [96]. To extend these observations, we transfected HEK293T cells with a plasmid co-expressing StrepII-tagged GIGYF2 and untagged EIF4E2 [95]. This plasmid produced functional protein, as evidenced by partial rescue of GIGYF2Δ and EIF4E2Δ reporter lines (S8A Fig). After 2 days, the transfected cells were lysed and tagged GIGYF2 was immunoprecipitated and subjected to mass-spectrometry analysis. As expected, EIF4E2 was highly enriched in the immunoprecipitated samples, and we also detected the NMD factor UPF1 (Fig 6A and S14 Table), as well as the EJC components EIF4A3 and MAGOHB, and to a lesser extent UPF3B and CASC3. Treatment of lysates with RNase A significantly reduced the interaction of GIGYF2 with UPF1 and the EJC components (Fig 6B and 6D), indicating that this interaction was largely mediated through mRNA contacts. These results are consistent with the idea that both proteins simultaneously bind to some mRNAs though it does not rule out a directly interacting population, or that one or both proteins need to be bound to mRNA or ribosomes to interact with one another. We reasoned that the mRNAs on which GIGYF2•EIF4E2 and UPF1 colocalize would be those with PTCs. To test this, we increased the abundance of cytoplasmic mRNAs with PTCs by treating the cells with spliceostatin A (SSA) for 16 hours prior to lysis and IP. Spliceostatin A inhibits the activity of splicing factor SF3b, leading to nuclear export and translation of intron-containing mRNAs [106], which almost always contain a PTC. Treatment with spliceostatin A led to a strong increase in association of UPF1 and UPF3B with GIGYF2 (Fig 6E). Intriguingly, spliceostatin A treatment also led to an increase in the association of SMG1 with GIGYF2, even more so than UPF1 and almost all other RBPs (Fig 6E). This result is consistent with the idea that GIGYF2 is directly associated with mRNAs in the process of being recognized for NMD, since SMG1 is thought to only transiently interact with NMD targets [25,34,107]. Given this result and the epistasis that we observed between SMG1i treatment and GIGYF2•EIF4E2 knockout, we asked whether the UPF1-GIGYF2•EIF4E2 interaction depended on the phosphorylation state of UPF1. We repeated the IPs in cells pre-treated with SMG1i to prevent UPF1 phosphorylation and found the RNA-dependent interaction to be unaffected by SMG1i treatment (S8 Fig and S15 Table). Together, these data provide evidence for the coordinate action of GIGYF2•EIF4E2 and UPF1 in repressing the translation of well translated PTC-containing mRNAs.

## Discussion

Here we have presented the results of a screen for genes that repress the levels or translation of mRNAs containing a premature translation termination codon. Our approach differed from previous screens for NMD factors in the use of a reporter with a long coding region as a 3′ UTR, instead of an intron-containing 3′ UTR. Our screen identified nearly all the previously known genes essential for mammalian NMD as well as a few unexpected genes. These included components of the EJC and the translational repressors GIGYF2 and EIF4E2. These findings led us to discover a novel role for GIGYF2•EIF4E2 in translational repression of a subset of NMD targets, providing a safeguard against the production of truncated proteins that could lead to dominant negative toxicity or proteotoxic stress.

### The role of the exon-junction complex in NMD the absence of 3′UTR splicing

While our screen identified many of the expected NMD factors, we were surprised to see a strong enrichment for guides against components of the EJC as well as the splicing factor AQR which is required for EJC deposition (Figs 2B and S3B) [108]. The increase in expression caused by depletion of these components is unlikely to be caused by a failure to splice the intron in the 5′ UTR of our reporter, as loss of this EJC would be expected to reduce nuclear export and translation of our reporter [109,110], leading to a decrease in tagBFP. Nor is it the result of cryptic splicing, as we were unable to detect splicing downstream of the premature termination codon (S3D and S3E Fig). These data raise the possibility that splicing-independent deposition of EJC factors contributes to NMD of the reporter as well as other cellular “EJC-independent” NMD targets without detectable splicing downstream of their stop codons. This hypothesis was supported by analysis of RNA-seq data from knockdowns of EJC components. It is also possible that the effects that we observed are due to decreases in the expression or function of NMD factors upon loss of EJC subunits. While our data suggest that the mRNA levels of NMD factors are increased upon EJC factor depletion, we have not ruled out a reduction in protein levels or post-translational modifications that could reduce the function of this pathway. Crosslinks between EJC components (including UPF3B) and RNA have been detected in unexpected mRNA locations [78,79], and artificial tethering of the EJC protein RBM8A downstream of a stop codon is sufficient to induce NMD [111]. These results indicate that EJC-dependent NMD could operate in the absence of splicing, but further testing is required to identify if this actually occurs *in vivo*.

### Translational repression of NMD targets

Our finding that GIGYF2•EIF4E2 represses translation of NMD targets is reminiscent of previous studies in yeast suggesting that NMD targets are translationally repressed [59] and their protein products targeted for degradation [112]. Work in mammalian cells suggested that phospho-UPF1 inhibits bulk translation through interactions with eukaryotic initiation factor eIF3 [60], but a specificity for NMD targets was not shown. In contrast, our study defines molecular mechanisms involved in specific translational repression of some PTC-containing mRNAs in mammalian cells, in a manner that depends on the presence of the PTC.

Our polysome analysis (S6D Fig) showed that GIGYF2•EIF4E2 does not affect bulk translation in K562 cells. Global increases in polysomes were previously observed in EIF4E2Δ mouse brains [84], though not in EIF4E2Δ mouse embryonic fibroblasts [102], indicating that some tissues and cell types may have more GIGYF2•EIF4E2-mediated translational regulation than others. This could reflect differences in substrate expression or the activity of the various pathways that recruit GIGYF2 and/or EIF4E2 for translational repression or mRNA decay [53–55,57,58,95–97,113]. In addition to these previously described pathways, we have now shown that some NMD targets are also targeted by this complex. For endogenous mRNAs, our data showed this effect to be limited to NMD targets with 3′ UTR introns (Fig 4B). This could mean that repression by GIGYF2•EIF4E2 is limited to the EJC-dependent subset of NMD targets. This is unintuitive given that our screen reporter lacked a 3′ UTR intron, but it would be consistent with our finding that this reporter still requires EJC components for repression. This specificity for transcripts with 3′ UTR introns is biologically consistent with the fact that they tend to encode potentially harmful nonfunctional proteins, so translational repression of these isoforms would be especially beneficial to the cell. We also noticed that EJC-dependent NMD targets had higher ribosome occupancy in our ribosome profiling data. Alternatively, the repressive effect of GIGYF2•EIF4E2 may be more evident for EJC-dependent NMD targets since they undergo stronger repression by NMD and are more likely to be direct NMD targets, and not indirect targets of NMD factor depletion. While our analysis was only able to identify 16 transcripts that were coregulated by SMG1i out of the 107 transcripts translationally repressed by GIGYF2 or EIF4E2, the number of affected transcripts is likely to be higher for both technical and biological reasons. First, it is difficult to detect translational changes of low-abundance NMD splice isoforms based on the short reads obtained from ribosome profiling. Second, the translational repression that we observe likely represents a transient intermediate preceding mRNA degradation by NMD or other pathways [53,56,57]; such intermediates are often invisible to steady-state methods like RNA-seq and ribosome profiling [9,114].

### Physical colocalization of GIGYF2•EIF4E2 and UPF1 on PTC-containing mRNAs

Our mass spectrometry data indicates that GIGYF2•EIF4E2 colocalizes with UPF1 on some mRNAs. The increased interaction that we observe upon spliceostatin A treatment, which causes an accumulation of PTC-containing mRNAs in the cytoplasm, further supports the model that these proteins colocalize on PTC-containing mRNAs. Since this interaction is sensitive to RNase, these proteins are either indirectly bound to the same mRNAs, or their binding to each other otherwise depends on being bound to RNA. The resistance of the interaction between UPF1 and GIGYF2•EIF4E2 to SMG1i treatment suggests that the translational de-repression of NMD targets by SMG1i is not due to dissociation of GIGYF2•EIF4E2 from UPF1. This also seems to be the case for the NMD endonuclease SMG6, which binds to the N-terminal region of UPF1 independent of UPF1’s phosphorylation state [38,115], though SMG6 activity must still be activated by the phosphorylation event. This contrasts with the exonucleolytic decay activators SMG5 and SMG7, which specifically bind to phosphorylated UPF1 [38,115].

Neither GIGYF2 nor EIF4E2 have been found in previous screens for NMD factors. This might be explained by the choice of reporters or cell lines used in previous screens. All 3 recent human NMD screens used intron-containing sequences from the human β-globin gene to induce NMD. Two of these reporters [28,29] used a UAG stop codon at β-globin codon 39, matching a naturally-occurring mutation found in β-thalassemia patients [35], while the third [30] used a UAA stop codon. Our reporter, on the other hand, used a long tdTomato coding sequence with a UGAC PTC to trigger NMD. Several reports suggested that the rate of translation termination or ribosome recycling is slower at PTCs triggered by long 3′ UTRs, possibly due to increased spatial separation between the terminating ribosome and polyA binding protein [42–45,111]. This raises the possibility that certain PTC stop codons are targeted by GIGYF2•EIF4E2 because they are particularly slow to terminate or recycle. As previous studies have shown a role for ribosome collisions in the recruitment of GIGYF2•EIF4E2 [55,95], it is tempting to speculate that collisions or other unusual ribosome conformations may play a role at PTC stop codons as well. It is also possible that the exons that encode PTCs, which have not been selected to efficiently encode protein, have poorly optimized stop codon contexts which increase the rate of ribosome collisions on these mRNAs, as we observed on our reporter.

What is the purpose of repressing translation of an mRNA that is marked for degradation? Translational repression by GIGYF2•EIF4E2 prevents loading of excess ribosomes onto a PTC-containing mRNA, reducing production of aberrant protein and the number of ribosomes that would require rescue after NMD-directed endonucleolytic cleavage by SMG6 [116]. Since several translation termination events occur on average before the initiation of NMD on an mRNA [103], an mRNA could be loaded with several ribosomes by the time NMD is triggered, and this loading could continue as the mRNA is degraded, since the mRNA’s 5′ cap is still intact. Repression would be most important for “degradative” targets of NMD which are more likely to encode truncated proteins, as opposed to the “regulatory” targets which generally encode functional proteins [46]. Notably *S*. *cerevisiae* UPF1 has been reported to mediate both translational repression [59] of NMD target mRNAs and proteasomal degradation of their protein products [112]. These processes are analogous to the quality control events triggered by ribosome collisions, which repress mRNA translation [55,95,98] and degrade ribosome-associated nascent peptides [117]. In the case of mRNAs with PTCs, repression by GIGYF2•EIF4E2 serves as a fail-safe NMD mechanism that reduces production of truncated peptides, thereby reducing the possibility of dominant negative phenotypes or proteotoxic stress.

## Methods

### Plasmids

Lentiviral expression plasmid pWPI (addgene #12254) was digested with restriction enzymes PmeI (NEB) and BstBI (NEB) and used for Gibson assembly with geneblock (IDT) fragments oBZ435 and oBZ437 to make pBZ102_lenti_tagBFP_2A_bamHI_tdTomato. To insert premature stop codons, pBZ102 was digested with BamHI. Pairs of complementary DNA oligos encoding the PTC context were annealed and inserted into the plasmid backbone with Gibson assembly (oBZ447-462 for normal stop codons, oBZ499-504 for contexts from [72]) to create plasmids pBZ103_UGGC, pBZ105_UGAC, pBZ147_AQP4, pBZ149_OPRL1. The pBZ280 plasmid, in which the PGP of the 2A peptide is mutate to AGA, was made by QuikChange mutagenesis (Agilent) of pBZ105. CRISPR guide plasmids were made by digesting lentiGuidePuro (addgene # 52963) with BsmBI and ligating in pairs of phosphorylated annealed oligos containing sgRNA sequence and appropriate overhangs [118]. pcDNA_4EHP-P2A-TwinStrep-GIGYF2 [95] was a kind gift from Ramanujan Hegde. Guide RNA target sites in this plasmid were mutated by QuikChange multi-site mutagenesis (Agilent) to create pBZ361 for rescue experiments. The GFP control plasmid (pBZ295) was made by cutting GFP-UPF1 plasmid with XhoI and NotI, blunting the ends with Phusion polymerase (NEB) and circularizing the products with T4 DNA ligase (NEB). Descriptions of oligos and plasmids are available in S16 and S17 Tables.

## Cell culture

Mammalian cell lines were grown in cell culture incubators at 37°C in the presence of 5% CO_2_. K562 cells were grown in Roswell Park Memorial Institute (RPMI) 1640 media (Gibco) supplemented with 10% FBS (Gibco), 2mM Sodium Pyruvate (Gibco) and 1x GlutaMAX (Gibco) or 2mM Glutamine (Gibco). Media without phenol red was used for flow cytometry assays. HEK293FT cells were cultured in Dulbecco’s Modified Eagle Media (DMEM) (Gibco) supplemented with 10% FBS (Gibco).

### Lentivirus production and infection

For lentivirus production 25,000 HEK293FT cells (7 or fewer passages) (ThermoFisher R7007) were plated in a well of a 96-well plate pre-coated with poly-D-lysine. The next day, cells in each well were co-transfected with 44 ng lentiviral transfer plasmid, 34 ng psPAX2 (Addgene #12260), and 20ng pMD2.G (Addgene #12259), using lipofectamine 3000 (ThermoFisher L3000008) following the manufacturer’s protocol. For larger scale production, cell number, plasmid and lipofectamine reagents were scaled by surface area. Virus was harvested at 48, 72, and 96 hours, as allowed by cell viability, and frozen or used immediately. For lentiviral CRISPR libraries, 7x15 cm dishes were used, and virus was filtered with an 0.45 micron bottle-top filter, and stored at 4°C until all timepoints were harvested, at which point all virus was pooled, concentrated with Centricon Plus 70 centrifugal filter (Millipore UFC10008) and flash-frozen in liquid nitrogen for storage at -80°C. K562 cells were lentivirally transduced using the spinfection method: 90,000 cells were seeded per well of a 96-well PCR plate in RPMI+10% FBS along with lentiviral supernatant and 8 μg/ml polybrene in a total volume of 50–100 μl. The plate was centrifuged at 33°C for 2 hours at 1000xg, and the cells were resuspended in fresh media. For titering assays, 1000 cells were plated per well of a black-walled clear-bottom 96-well (Corning 3603) plate in quadruplicate 2 days after infection, treated with puromycin (or other selection drug) and assayed after 3 more days using the CellTiter-Glo viability assay (Promega) following the protocol of [118], in a Biotek Synergy H1 plate reader.

### Generation of reporter cell lines

Lentiviral reporter vectors were integrated into K562 cells as described under “lentivirus production and infection”. Monoclonal lines were then isolated by FACS, by selecting cells with increased tagBFP signal, and tdTomato signal for no-PTC or high readthrough lines. PTC-containing monoclonal lines were further screened by microscopy and flow cytometry to identify lines with a robust and uniform increase in tagBFP and tdTomato in response to G418 treatment. For CRISPR experiments, these lines were then further transduced with lentiCas9-Blast (Addgene 52962) and, after 2 days of recovery, treated with 15 μg/ml blasticidin (ThermoFisher A1113903) for a week and allowed to recover for 3 days before performing experiments.

### Flow cytometry

Analyses in Figs 1 and S1, S2, S3B and S3C were performed on a BD LSRII flow cytometer with 405 nm excitation, 450/50 nm emission filter for tagBFP and 488 nm excitation, 575/26 emission filter for tdTomato. For LSRII use, cells were filtered into a Corning Falcon 352235 cell trainer tube. All other flow cytometry assays were performed on a Luminex Guava EasyCyte HT with 405 nm excitation, 450/45 nm emission filter for tagBFP and 532 nm excitation, 620/52 emission filter for tdTomato. For G418 treatment, media was supplemented with HEPES pH7.0 (Gibco) to 10mM to neutralize the acidity of G418 (ThermoFisher 10131035). For all experiments, events were gated on their side and forward scatter to generate a uniform cell population, and to remove debris, 2-cell events and dead or sick cells.

### Northern blotting

Northern blotting was performed as in [65], with some modification. 3 million K562 cells were transferred to fresh media with or without SMG1i for 4 hours. Cells were pelleted, washed with PBS, and resuspended in 300ul TRIzol (ThermoFisher 15596026). RNA was isolated using the Zymo Direct-zol RNA miniprep kit (Zymo Research R2050) and quantified by A260 absorbance. 8 μg of RNA per sample was denatured at 70°C for 10 minutes and separated on a 0.7% agarose MOPS-formaldehyde gel, alongside RiboRuler High Range RNA ladder (ThermoFisher SM1821). RNA was transferred to a Hybond N+ (Cytiva RPN303B) membrane using wet vacuum transfer for 4 hours. After UV crosslinking 3 times in a StrataLinker using auto setting, the membrane was stained with methylene blue and positions of ladder and rRNA bands were marked. Membranes were hybridized overnight in roller bottles at 42°C in Rapid-Hyb buffer (Cytiva RPN1635) along with 50 picomoles of oBZ732 5′ end-labeled with ^32^P. Membranes were then washed 3x for 20 minutes at 50°C in (2xSSC, 0.1% SDS), and exposed to a phosphor screen for 24 hours, followed by scanning on a Typhoon FLA9000. Bands were quantified using ImageQuant TL (Cytiva).

### CRISPR screening

The 2-vector GPP Brunello CRISPR library (Addgene 73178) was propagated by electroporation using the method of [118], grown on 25 cm plates, and purified using Qiagen megaprep kit (Qiagen 12181). For screening, 200 million cells were infected as in “lentivirus production and infection” with CRISPR library in duplicate in 6-well dishes in 99ml media and 1ml lentiviral concentrate. After spinfection, cells were combined, pelleted, and resuspended in 400ml fresh media in T225 flasks (Corning). On subsequent days, cells were diluted to 500,000 cells/ml with fresh media to maintaining 350 million cells per replicate, freezing down remaining cells. 2 days after infection, cells were set aside for titering and puromycin was added to 0.75 μg/ml. The titers indicated an infected fraction of 30%, corresponding to approximately 60 million infected cells per replicate. 6 days post infection, FACS sorting was performed on a BD Aria IIu with an 85 micron nozzle, 405 nm excitation, 450/40 nm emission filter for tagBFP and 488 nm excitation, 575/26 emission filter for tdTomato. The top and bottom 10% of cells were simultaneously collected from a sort of 50 million cells resulting in ~1.5 million cells collected per direction per replicate, which were pelleted, washed in PBS, and frozen in liquid nitrogen. 70–90 million unsorted cells were pelleted and frozen in liquid nitrogen as an input control. Library preparation was carried out in a dedicated space free of purified CRISPR plasmids or amplified libraries. DNA was extracted using Zymo quick-gDNA midiprep kit (Zymo Research D3100) for input or miniprep kit (Zymo Research D3024) for sorted cells, with these modifications: Proteinase K treatment was done overnight, and lysate was treated with 67ug of RNase A (ThermoFisher ENO531) per ml of lysate. Extracted DNA was quantified by nanodrop, and 50 μl of PCR was set up per 5ug of DNA as follows: 25 μl NEBNext Ultra II Q5 Master Mix (NEB M0544S) 1.25 μl of a 10 μM equimolar mix of oBZ475-oBZ478 staggered forward primers, 1.25 μl of 10 μM barcoded reverse primer (oBZ479-oBZ490), gDNA, water to 50 μl. The PCR mixture was aliquoted into strip tubes or 96-well plates and cycled: 98°C for 3 minutes, 19–21 cycles of (98°C for 10 seconds, 63°C for 10 seconds, 72°C for 25 seconds), 72°C for 2 minutes. PCR reactions for each sample were then pooled, and 200–300 μl of each was subjected to SPRIselect (Beckman Coulter B23318) cleanup with 0.55x and 0.95x cuts. A 1:10 or 1:100 dilution of each library was quantified by Agilent Bioanalyzer High-sensitivity DNA chip (Agilent 5067–4626), and libraries were pooled, including a 2-fold higher molarity of input libraries compared to sorted libraries. Libraries were subjected to 66 bp single-end sequencing on an Illumina Hiseq 2500 with a 5% PhiX spike-in. Due to a processing accident, one replicate of low-ratio cells for the AQP4 reporter had very low read counts.

### Analysis of CRISPR screening data

Guide spacers were counted from sequencing data using a modified version of count_spacers.py from https://github.com/fengzhanglab/Screening_Protocols_manuscript [118]. Guide counts were used to run MAGeCK [76]. Sequencing FASTQ files have been deposited in the NCBI sequence read archive (PRJNA675708), and analysis code deposited at https://github.com/borisz264/NMD_screen_2020.

### CRISPR validation

For large-scale validation, the top 2 guides for each gene being validated were selected based on their enrichment p values and cloned into lentiGuidePuro as described above. For viral transduction (As in S2B and S2C Fig), lentiviral supernatant was generated in 96-well plates and directly used to infect reporter K562 cells as described above. Cells were treated with 0.75 μg/ml puromycin after 2 days and assayed by flow cytometry after 2–7 more days. For small-scale electroporation experiments (as in Fig 2C), 200,000 cells were electroporated with 1000ng of lentiviral sgRNA plasmid in a volume of 20ul using the Lonza SF Cell Line 4D-Nucleofector X Kit S (Lonza V4XC-2032) according to the manufacturer instructions and resuspended in 3 ml of media without phenol red. Cells were treated with 1 μg/ml puromycin the next day and assayed by flow cytometry over subsequent days.

### Nanopore direct RNA sequencing

A 10cm dish of HEK293FT cells was transfected with 15 μg of UGAC (pBZ105) or AQP4 (pBZ147) reporter plasmid. After 24 hours, the media was aspirated, and cells were wash with PBS and resuspended in 1ml of TRIzol (ThermoFisher). RNA was extracted using direct-zol RNA miniprep kit (Zymo Research R2050) and ~30–40 μg was polyA-selected with the NEBNext poly(A) mRNA magnetic isolation module (NEB E7490S) following the manufacturer’s instructions, except for the increased RNA input and the use of a 3x volume of beads. The entirety of the polyA-selected RNA (~500ng) was used to prepare direct RNA sequencing libraries (Oxford Nanopore SQK-RNA002), which were sequenced with a nanopore Minion using an R9.4.1 flow cell (FLO-MIN106D R9 Rev D). Reads were mapped to plasmid sequences using FLAIR (https://github.com/BrooksLabUCSC/flair) [119], and alignments parsed using Pysam (https://github.com/pysam-developers/pysam) [120] and custom code available at https://github.com/borisz264/NMD_screen_2020. Raw sequencing reads have been deposited in the NCBI sequence read archive under BioProject ID PRJNA675956.

### Genotyping of knockout lines

Genotyping on polyclonal and monoclonal lines was performed based on the method of [118]. K562 lines were pelleted and lysed with QuickExtract solution (Lucigen) following the manufacturers protocol. Targeted regions were PCR amplified using NEBNext Ultra II Q5 Master Mix (NEB M0544S) with 20 cycles and 60°C annealing. For GIGYF2, an equimolar mix of forward primers oBZ706/oBZ707 were used with reverse primer oBZ708. For EIF4E2, oBZ709/oBZ710 and oBZ711 were used. PCR reactions were cleaned up with SPRIselect (Beckman Coulter B23318) at 0.8x ratio, and a second round of 10 PCR cycles was performed on this product using different combinations of primers oBZ712-oBZ731 to add multiplexing barcodes and Illumina adaptor sequences. After SPRIselect cleanup, each library was quantified by Agilent Bioanalyzer High-sensitivity DNA chip (Agilent 5067–4626), and libraries were pooled with an estimated 20x molar excess of polyclonal samples to monoclonal and subjected to 300nt single-end sequencing on an Illumina MiSeq, using 300 cycle nano kit, at the Johns Hopkins Medicine Transcriptomics and Deep Sequencing Core. Demultiplexed reads were analyzed for indels using a modified version of calculate_indel.py from https://github.com/fengzhanglab/Screening_Protocols_manuscript [118] and custom python code.

### Western blotting

For K562 experiments, 2–5*10^5^ cells were treated with SMG1i for 24 hours in a 6-well dish. HEK293FT cells were harvested by scraping. Cells were pelleted at 500xg for 2 minutes. Cell pellet was resuspended in 30 μl ice-cold lysis buffer (50mM HEPES pH7.4, 150mM KOAc, 15mM MgOAc_2_, 1% triton, leupeptin, pepstatin, PMSF, 1x EDTA-free Complete (Sigma 11873580001), 2 U Turbo DNase/ml (ThermoFisher AM2238), then gently pipetted 10 times to lyse. After 5 minutes on ice, lysates were clarified by centrifugation at 20,000xg for 10 minutes at 4°C and supernatant transferred to a new tube. For S4F Fig, samples were electrophoresed in a 4% SDS-PAGE Bis-Tris gel, transferred to a nitrocellulose membrane, and blotted with a 1:10,000 dilution of ZNF598 antibody (Bethyl A305-108A) and 1:50,000 β-actin (R&D MAB8929). For S1B Fig membrane was blotted with 1:5,000 tagRFP antibody (Thermo R1037) and 1:5000 GAPDH antibody (abcam ab9482). After incubation with LI-COR secondary antibodies, blots were scanned on LI-COR imager. For S5F Fig a 1:10 dilution of each sample was used for blotting with Jess automated western blotting instrument (proteinsimple) following manufacturer’s instructions. TagRFP (evrogen AB233) and β-actin (R&D MAB8929) primary antibodies were used at 1:200 dilution. Anti-rabbit chemiluminescent and anti-mouse near-infrared secondary antibodies (simplewestern) were used. Peak areas for β-actin were used to normalize the peak areas for tagBFP in each sample, which was then further normalized to the untreated WT sample for that day.

### Polysome gradients

5*10^8^ cells were lysed in 250ul ice-cold lysis buffer (50mM HEPES pH7.4, 150mM KOAc, 15mM MgOAc_2_, 1% triton, 2 U Turbo DNase/ml (ThermoFisher AM2238), 1mM DTT, 2U/ml superasin) then gently pipetted 10 times to lyse. After 5 minutes on ice, lysates were clarified by centrifugation at 20,000xg for 10 minutes at 4°C and supernatant transferred to a new tube. Samples were loaded onto a 10–50% sucrose gradient in (50mM HEPES pH7.4, 150mM KOAc, 15mM MgOAc_2_, 1% triton, 1mM DTT), and centrifuged for 2 hours at 38,000 RPM in an SW-41 rotor. Gradients were fractionated with in-line UV monitoring using a BioComp piston fractionator.

### RNA-seq and ribosome footprint profiling library preparation

Our ribosome profiling protocol was adapted from [121]. 3 million cells per replicate were resuspended in fresh media, and 3 μl 1mM SMG1i or 3ul DMSO was added and incubated for 4.5 hours in a T25 flask. Cells were harvested by centrifugation at 500xg for 2 minutes at room temperature, media aspirated, and resuspended in 200 μl of mammalian footprint lysis buffer [20 mM Tris-Cl (pH8.0), 150 mM KCl, 5 mM MgCl2, 1 mM DTT, 1% Triton X-100, 2 units/ml Turbo DNase (Thermo Fisher), 0.1 mg/mL cycloheximide (Sigma-Aldrich)]. Lysates were incubated on ice for 10 minutes and then clarified by centrifugation at >15,000*g* for 10 minutes. For RNA-seq, RNA was purified from an aliquot of lysate using Zymo RNA microprep kit, and libraries prepared using the Zymo-Seq RiboFree Total RNA Library kit (Zymo Research R3000) with 1.5 μg of total RNA as input. For ribosome profiling, 0.5 A260 units of lysate was digested with 750 units of RNase I (Ambion) in 300 μl lysis buffer at 25°C for 1 hour. Reactions were quenched with 200 U SUPERase-in (Thermo Fisher) and pelleted over a sucrose cushion for 1 hr at 100,000 RPM. Pellets were resuspended in TRIzol (Thermo Fisher) and RNA extracted with the Zymo Direct-zol RNA miniprep kit. Fragments were size selected on a 15% TBE-urea PAGE gel (Bio-Rad), cutting between 20–34 nt size markers. After gel elution, fragments were dephosphorylated, ligated to preadenylated 3′ linker oBZ407, reverse transcribed using protoscript II (NEB) and RT primer oBZ408, and RNA degraded by base hydrolysis. cDNA was depleted of rRNA sequences by adding 1 μl of a 12 μM (total) equimolar mix of oBZ784-793, 1 μl 20x SSC, to 8 μl cDNA in water. Mixture was annealed by heating to 90°C, then cooling to 37°C at 0.1°C/second. Biotinylated oligos were captured on 27.5 μl of MyOne Streptavidin C1 dynabeads (ThermoFisher) and incubated at 37°C for 15 min with shaking at 500 rpm in a thermomixer. After incubation, beads were magnetically precipitated, supernatant was collected, and isopropanol precipitated to recover RNA. Depleted cDNA was gel purified, circularized with Circ Ligase (Lucigen), and PCR amplified. Amplified libraries were quantified by bioanalyzer high-sensitivity DNA chip (Agilent) then pooled and sequenced on an Illumina HiSeq 2500 with 150nt paired-end reads (GENEWIZ).

### Ribosome footprint profiling and RNA-seq data analysis

Demultiplexed ribosome footprint reads (only read 1 was used) were trimmed of the 4 random nt from the 5′ end with seqtk (https://github.com/lh3/seqtk), then trimmed of 3′ adaptor sequence (NNNNNNCACTCGGGCACCAAGGAC) using Skewer [122]. For RNA-seq datasets, AGATCGGAAGAGCACACGTCTGAACTCCAGTCA was trimmed from read 1, and AGATCGGAAGAGCGTCGTGTAGGGAAAGAGTGT from read 2. In order to select a set of transcripts to use for further analysis, we pooled all of our ribosome profiling libraries into a single dataset and ran ORFquant [99] with the Homo Sapiens GENCODE 35 genome and annotations as a reference. We filtered out transcripts without an annotated stop codon, and for all sets of transcripts that shared a stop codon, we chose a single transcript with the longest CDS and then, in case of a tie the longest transcript overall. We added our reporter transcript, and used the resulting transcript set for further analysis. Salmon [100] was used to estimate RNA-seq and ribosome footprints read counts, mapping to whole transcripts, or coding regions, respectively. We used the Xtail package [101] to compute ribosome occupancy fold changes and statistical significance.

To empirically identify NMD target transcripts, we used Salmon to quantify read counts for our transcript set based on the RNA-seq datasets from [82]. We then used DESeq2 [123] to quantify transcript fold changes and statistical significance for knockdown and rescue of NMD factors. We then combined the Benjamini-Hochberg corrected p-values from this analysis into a single value using a combination of Fisher’s method and sum of p-values as described in [82], and used a cutoff of 0.05 to select NMD target transcripts. Further analysis and plotting were done using custom Python code available at https://github.com/borisz264/NMD_screen_2020. Sequencing data have been deposited in the NCBI sequence read archive (PRJNA675773).

To analyze the data from [57], ribosome profiling and RNA-seq reads were downloaded from GEO (accessions GSE144841 and GSE149279), and processed as above, except using 3′adaptor NNNNTGGAATTCTCGGGTGCCAAGG, and mapped to the same sets of transcripts identified in our own data.

For analysis of ENCODE data, RNA-seq data for experiment series ENCSR263EJV (HepG2 EIF4A3 shRNA), ENCSR453OOG (HepG2 MAGOH shRNA), ENCR733XTT (K562 EIF4A3 shRNA), ENCSR726JGY (K562 MAGOH shRNA) were downloaded and mapped to the transcript sets identified above using Salmon.

### StrepII-GIGYF2•EIF4E2 coimmunoprecipitation

HEK293T cells were seeded at 3 million cells per plate and allowed to grow for 24 hours. At 24 hours cells were replenished with fresh DMEM supplemented with 10% FBS, transfected with 10 μg pcDNA3.1 4EHP-2A-StrepII-GIGYF2 (kind gift from Ramanujan Hegde), and allowed to grow for 48 hours. Cells were replenished with fresh media 24 h after transfection. 16 h prior to harvest, cells were treated with SSA at 100 ng/ml. For SMG1i treatment, cells were treated with SMG1i (300 nM) for 4 h prior to harvest. Cells were washed with warm PBS (containing 360 μM emetine) and lysed in 300 μl ice-cold lysis buffer (50 mM HEPES pH 7.4, 100 mM KOAc, 15 mM Mg(OAc)_2_, 5% Glycerol) supplemented with 0.5% Triton-X-100, 360 μM emetine, 20 nM avidin, 1 mM TCEP, 1x phosphatase inhibitor cocktail (Cell Signaling Technology), 10 mM N-ethylmaleimide, 2x cOmplete EDTA-free Protease Inhibitor Cocktail tablets (Roche), 1 mM PMSF (Sigma), 1x mammalian protease inhibitor cocktail (Sigma) and 8 units/ml Turbo DNase (Thermo Fisher). Cell lysates were clarified in a tabletop micro-centrifuge (7000 x g, 5 minutes, 4°C) and equal protein amounts from the clarified supernatant (determined by BCA assay) were used for each immunoprecipitation. For RNase A treatment, clarified cell lysates were treated with RNase A (Ambion) using the following condition– 1 μg RNase A was added per 100 μg total RNA in the supernatant and shaken at 500 rpm (20 min, 25°*C*) on a table-top thermo-mixer; the reaction was quenched by the addition of SUPERase•In RNase A inhibitor (~200 units per 100 μg RNA). Clarified supernatants for the different conditions were incubated with MagStrep “type 3” XT beads (IBA Life Sciences) for 1 h (4°C) with gentle rocking as per manufacturer guidelines. Following incubation, samples were bound to Dyna-Mag magnet as per manufacturer guidelines, the flow-through was removed, and the samples were washed with lysis buffer supplemented with 0.5% Triton-X-100, 360 μM emetine (4 washes x 10 min, 4°C), followed by 4 washes (10 min, 4°C) with lysis buffer not containing glycerol or detergent. Proteins were eluted from the beads using Buffer BXT (100 mM TRIS pH 8.0, 150 mM NaCl, 1 mM EDTA, 50 mM biotin, IBA Life Sciences) and processed for MS.

### Protein digestion

Protein extracts (~10 μg) were diluted up to 300 μl in 10 mM triethyl ammonium bicarbonate (TEAB) and were reduced with 15 μl of 7.5 mg/ml DL-dithiothreitol (DTT) (60°C, 1 hour). After cooling to room temperature, samples were alkylated with 15 μl of 18.5 mg/ml iodoacetamide for 15 minutes at room temperature in the dark. Reduced and alkylated proteins were buffer-exchanged on a 30 kDa molecular weight spin cartridge (Amicon Ultra 0.5 ml, Millipore Sigma) and washed four times with 400 μl 10 mM TEAB. Proteins were digested overnight at 37°C on the filter with 300 μl Trypsin (20 μg in 3 ml 10 mM TEAB, Promega Sequencing Grade Modified Trypsin). Additional Trypsin (100 μl of 10 mg/ml) was added the next morning (37°C, 1 hour). Peptides were removed from the top of the filter and the filter was washed twice with 300 2% acetonitrile, 0.1% formic acid. All washes were combined and dried.

### Liquid chromatography and mass spectrometry

Peptides were analyzed by liquid chromatography interfaced with tandem mass spectrometry (LC/MS/MS) using an Easy-LC 1000 UPLC system (Thermo Fisher) interfaced with an Orbitrap Q-Exactive Plus Mass Spectrometer (Thermo Fisher). Fractions were resuspended in 20 μl loading buffer (2% acetonitrile in 0.1% formic acid) and analyzed by reverse phase liquid chromatography coupled to tandem mass spectrometry. Peptides (25%, approx. 0.5μg) were loaded onto a C18 trap (S-10 μM, 120 Å, 75 μm x 2 cm, YMC, Japan) and subsequently separated on an in-house packed PicoFrit column (75 μm x 200 mm, 15u, +/-1 μm tip, New Objective) with C18 phase (ReproSil-Pur C18-AQ, 3 μm, 120 Å, www.dr-maisch.com) using 2–90% acetonitrile gradient at 300 nl/min over 120 min. Eluting peptides were sprayed at 2.0 kV directly into the Q-Exactive Plus. Survey scans (full MS) were acquired from 350–1800 m/z with data-dependent monitoring with a loop count of 15. Each precursor individually isolated in a 1.4 Da window and fragmented using HCD activation collision energy 28 and 15 sec dynamic exclusion, first mass being 120 m/z. Precursor and the fragment ions were analyzed at resolutions 70,000 and 35,000, respectively, with automatic gain control (AGC) target values at 3e6 with 50 ms maximum injection time (IT) and 1e5 with 100 ms maximum IT, respectively. The mass spectrometry data have been deposited to the ProteomeXchange Consortium via the PRIDE partner repository with dataset identifiers PXD027479 and PXD027487.

### Mass spectrometry data analyses

Raw data was processed and analyzed using the MaxQuant (1.6.7.0) software suite [124]. Default settings were used except that ‘Match between runs’ was turned on to transfer peptide identification from an LC-MS run, in which the peptide has been identified by MS/MS, to another LC-MS run, in which no MS/MS data for the same peptide was acquired or no peptide was assigned [124]. Search parameters were as follows: a maximum of two missed cleavages were allowed, cysteine carbamidomethyl was included as a fixed modification, and variable modifications included oxidation of methionine, protein N-terminal acetylation, deamidation of glutamine and asparagine, and phosphorylation on serine, threonine, or tyrosine. Trypsin was used as the digestion enzyme, and the minimal peptide length was set to 7 amino acids. Searches were performed using a 20-ppm precursor ion tolerance for total protein level analysis. Database search was performed with Andromeda against Uniprot human database (UP000005640_9606.fasta; downloaded on 09/10/2018) with common serum contaminants and enzyme sequences. False discovery rate (FDR) was set to 1% at peptide spectrum match (PSM) and protein level. Minimum peptide count required for protein quantification was set to two. Protein groups were further analyzed using the Perseus [124]. Common contaminants, reverse proteins and proteins only identified by site were filtered out. LFQ values were transformed to log_2_ space and intensity distributions were checked to ensure that data was normally distributed.

### Electroporation rescue experiments

For GIGYF2/EIF4E2 rescue experiments 200,000 cells were electroporated at small scale with 1000ng of rescue plasmid (pBZ295-GFP or pBZ361-GIGYF2-EIF4E2) as described above and analyzed by flow cytometry after 2 days. All rescue plasmids were mutagenized to prevent cutting by the EIF4E2/GIGYF2 CRISPR guides used in the same experiment. All such mutations did not alter the encoded protein sequence.

## Supporting information

S1 Fig Validation of fluorescent protein choice and 2A peptide function of reporter. **A)** Flow cytometry of HEK293FT cells lentivirally transduced with indicated plasmids. tagBFP-tdTomato is the full reporter in Fig 1A with a UGG tryptophan codon at the PTC postion (Same as S1B Fig). **B)** Western blot of lysates from HEK293FT cells transduced with lentivirus containing reporters with the indicated stop codons, or UGG codon encoding tryptophan. Serial dilutions of UGG codon are also included.
(PDF)

S2 Fig Validation of NMD and readthrough measurements with reporter. A) Flow cytometry of monoclonal K562 cells expressing reporter with different stop codon contexts (or a UGG tryptophan codon). B) Flow cytometry of monoclonal K562 cells expressing reporter with AQP4 stop context PTC, treated with SMG1i or G418 for 24 hours. C) Same as panel B, for OPRL1 PTC reporter. D) Histograms of tdTomato/tagBFP signal for flow cytometry data from Fig 1E and 1F.
(PDF)

S3 Fig Validation of select screen hits. A) Volcano plot of fold enrichment and false discovery rate for CRISPR guides sequenced from cells with decreased tdTomato/tagBFP ratio compared to the input population for the AQP4 reporter. B) Volcano plot of fold changes and adjusted t-test p values for validation of select hits with the UGAC reporter. Each point is the relative mean tagBFP fluorescence (with average of nontargeting set to 1) of 2 or 3 separate lentiviral infections each with 2 separate guides (except for UPF1 and ETF1 controls, for which a single guide was used). Each t-test compares these 4 or 6 values against the 6 values for the nontargeting guides (2 separate guides in 3 separate infections). See S3 Table for individual values. The mean values include all cells in the sample, and therefore are affected by variations in transduction efficiency, CRISPR efficiency and knockout viability in addition to the magnitude of the effect on the reporter from the mutation. C) Histograms of tagBFP (blue line) or tdTomato (vermilion line) signal from flow cytometry of reporter lines lentivirally transduced with nontargeting guide (gray dotted line) or targeting guide (solid line), for reporter lines containing UGAC stop codon, AQP4 stop context, or no PTC (UGGC). SNRPF and AQR are splicing factors, RBM8A and EIF4A3 are core components of the EJC, and RPLP0 is a protein of the large ribosomal subunit. These samples were measured at 4 days post infection. D) Coverage of sequencing reads mapping to reporter from nanopore direct RNA sequencing of HEK293FT cells transfected with UGAC or AQP4 reporter plasmid. Fraction of reads at a position with a GU-AG splice junction are indicated as arcs, for introns with at least 2 supporting reads and making up at least 1% of reads spanning the intron ends. Reporter diagram indicates tagBFP and tdTomato coding regions as well as start and stop codons. E) Coverage of uniquely mapping paired-end RNA-seq reads mapping to integrated reporter in K562 reporter line treated with DMSO or SMG1i. Number of reads at a position with a GU-AG splice junction are indicated as arcs, for introns with at least 5 supporting reads. The density increases from 5′ to 3′ because nanopore direct RNA sequencing starts from the polyA tail and drops off as it proceeds to the 5′ end. The 3′ UTR region shares sequence with the Cas9:Blast transgene in these lines, causing some RNA-seq reads from that transgene to spuriously map here and likely causing a dampened apparent response to SMG1i treatment in this region.
(PDF)

S4 Fig Analysis of EJC component knockdown RNA-seq from ENCODE. A) Box plot of fold changes in mRNA levels for EIF4A3 or MAGOH knockdowns in HepG2 or K562 cells from ENCODE. Transcript sets are divided into all transcripts, empirically determined NMD targets with or without uORFs, and the subset of these NMD targets with 3′ UTR introns. Transcript counts for each set are indicated at left, and p values from Mann-Whitney U-test are indicated. +/- indicates that transcripts both with and without uORFs are included in the transcript set. B) Transcripts per million (TPM) from ENCODE RNA-seq data in Hep G2 cells for a subset of transcripts encoding NMD factors. T-test p values are indicated as asterisks. C) Same as panel B, for K562 cells. Note that in this panel, control 1 is the matched control for eIF4A3 knockdown, while control 2 is the matched control for MAGOH knockdown.
(PDF)

S5 Fig The effect of GIGYF2/EIF4E2/RACK1 on the reporter does not depend on ZNF598, EDF1 or the 2A peptide sequences. A) As in Fig 2C, with guides against EDF1 added. Note that all samples except EDF1 are the same ones as in Fig 2C, as these electroporations were performed simultaneously. B) tagBFP signal from flow cytometry of reporter lines transduced with nontargeting guide (NT1) or guides against indicated genes for reporter line containing UGAC stop codon. These samples were measured at 6 days post infection. C) Western blot of cell lines expressing nontargeting guides or a guide against ZNF598 from the same infections used for S3B Fig. Lysates were collected on the same day that the flow cytometry was performed. D) TagBFP intensities for independently generated reporter cell line with the 2A peptide upstream of the PTC disabled by mutating the terminal PGP residues to AGA. Electroporation and flow cytometry were performed in parallel with samples in panel A. Note that differences in signal intensity between the lines are likely due to differences in integration site and number, as well as stochastic variation in the monoclonal selection process.
(PDF)

S6 Fig Generation and characterization of GIGYF2/EIF4E2 knockout lines. A) Histograms of tagBFP signal from flow cytometry of UGAC reporter lines infected with nontargeting guide (dotted gray line) or targeting guide (solid blue line) 42 days after infection, and 12 days after sorting for 5000 cells with increased tagBFP signal. The polyclonal populations expressing GIGYF2 and EIF4E2 guides were used for further experiments, and for production of monoclonal lines. B) tagBFP signal from WT UGAC reporter line, and GIGYF2Δ or EIF4E2Δ polyclonal or monoclonal lines. C) Allele frequencies in monoclonal or polyclonal GIGYF2Δ and EIF4E2Δ lines, as assessed by high-throughput sequencing of the CRISPR target site. D) (left)UV absorbance trace from 10–50% sucrose density gradient fractionation of WT and GIGYF2Δ or EIF4E2Δ polyclonal lines. (right) Quantification of ratio of polysome area to 40S+60S+80S area (normalized to WT sample processed on same day) from 2 independent replicates. E) Median tagBFP intensities for indicated WT or EIF4E2Δ/GIGYF2Δ cell lines, either untreated (black circles), or treated with SMG1i for 24 hours (green circles). Biological replicate treatments were performed on 3 separate days. F) Quantification of automated western blotting of tagBFP relative to β-actin, for indicated WT or EIF4E2Δ/GIGYF2Δ cell lines, either untreated (black circles), or treated with 300nM SMG1i for 24 hours (green circles). Biological replicates were collected and blotted on 2 separate days and normalized to the untreated WT sample within each day. These samples are distinct from the ones used for flow cytometry. Dotted lines show the means for the untreated and SMG1i treated WT samples. Error bars show standard deviation. P value computed by two-tailed ratio paired t-test. All unmarked comparisons had p>0.05.
(PDF)

S7 Fig Ribosome occupancy changes in GIGYF2Δ or EIF4E2Δ cells. A) Box plot of fold changes in mRNA levels, ribosome footprints and ribosome occupancy (ratio of ribosome/mRNA) in EIF4E2Δ (green), GIGYF1Δ/GIGYF2Δ (blue), or ZNF598Δ HEK293T cells, relative to WT cells, for all transcripts (dark bars), empirically determined NMD targets (faded bars), and the subset of these targets with 3′ UTR introns (faded hatched bars). Transcript counts for each set are indicated at left, and p values from Mann-Whitney U-test are indicated. Raw data are from [57]. B) Comparison of fold changes in mRNA levels with fold changes in ribosome footprints for all quantified transcripts. Transcripts passing false discovery rate cutoff of 0.1 for changes in ribosome occupancy are shaded in black. Transcripts mentioned in the text are labelled in green. C) Venn diagram of transcripts passing cutoff from Fig 5A. P values for overlaps were computed by hypergeometric test, using a background set of 9,735 transcripts with computable fold changes in all datasets. D) Cumulative distribution of ribosome densities in EIF4E2Δ cells for NMD targets without (-EJC, solid orange line) or with (+EJC, dashed orange line) 3′ UTR introns. The number of transcripts in each set, and p value for Mann-Whitney U test against all transcripts (black line) is indicated in parentheses. The vertical black dotted line indicates the ribosome occupancy of the UGAC reporter. E) Same as panel D, but for the transcripts with increased ribosome occupancy in EIF4E2Δ (green line) or GIGYF2Δ (blue line).
(PDF)

S8 Fig Physical interaction between UPF1 and GIGYF2•EIF4E2. A) Flow cytometry analysis of rescue of EIF4E2Δ or GIGYF2Δ UGAC reporter polyclonal lines by electroporation of the GIGYF2:EIF4E2 coexpression plasmid used in Fig 6A–6E, with CRISPR guide sites mutated. B) Mass spectrometry analyses of StrepII-GIGYF2 immunoprecipitates with or without SMG1i treatment. Scatter plot showing log2(LFQ) intensity of proteins identified under SMG1i (y-axis) and untreated (x-axis) regimes. Values are from a single replicate of each sample.
(PDF)

S1 Table UGAC NMD screen results. Ranking, average log2 enrichments and false discovery rates for each gene in the UGAC NMD screen, as computed by MAGeCK.
(TXT)

S2 Table AQP4 NMD screen results. Ranking, average log2 enrichments and false discovery rates for each gene in the AQP4 NMD screen, as computed by MAGeCK.
(TXT)

S3 Table Validation of select screen hits by lentiviral infection and flow cytometry. Up to 2 guides per gene were used, in up to 3 replicates. Each dated column represents the average log2 tagBFP fluorescence for cells infected with that guide, normalized to the average of 2 nontargeting guides (NT). Blank cells mean that no infection for that guide was performed on that day. For each gene, the average of all days and guides is computed, as well as the t-test p value compared to the nontargeting guides, the rank of that gene by p-value, the Benjamini-Hochberg corrected p-value, and the -log of that value, which was used for plotting S2B Fig.
(TSV)

S4 Table Sequencing library sizes. Counts of transcript-mapping reads from ribosome profiling and RNA-seq datasets, as obtained from Salmon output.
(TXT)

S5 Table Read counts for all ribosome profiling and RNA-seq datasets for all transcript isoforms, as computed by Salmon. (TSV)

S6 Table TPMs (transcripts per million) for all ribosome profiling and RNA-seq datasets for all transcript isoforms, as computed by Salmon. (TSV)

S7 Table Log2 fold changes in RNA-seq (mRNA) and ribosome footprints (RPF), as well as ribosome occupancy (TE), and corresponding p values (pvalue.adjust) for all transcripts as computed by Xtail. For EIF4E2Δ vs WT in DMSO.
(TSV)

S8 Table Log2 fold changes in RNA-seq (mRNA) and ribosome footprints (RPF), as well as ribosome occupancy (TE), and corresponding p values (pvalue.adjust) for all transcripts as computed by Xtail. For EIF4E2Δ vs WT in SMG1i.
(TSV)

S9 Table Log2 fold changes in RNA-seq (mRNA) and ribosome footprints (RPF), as well as ribosome occupancy (TE), and corresponding p values (pvalue.adjust) for all transcripts as computed by Xtail. For EIF4E2Δ in SMG1i vs WT in DMSO.
(TSV)

S10 Table Log2 fold changes in RNA-seq (mRNA) and ribosome footprints (RPF), as well as ribosome occupancy (TE), and corresponding p values (pvalue.adjust) for all transcripts as computed by Xtail. For GIGYF2Δ vs WT in DMSO.
(TSV)

S11 Table Log2 fold changes in RNA-seq (mRNA) and ribosome footprints (RPF), as well as ribosome occupancy (TE), and corresponding p values (pvalue.adjust) for all transcripts as computed by Xtail. For GIGYF2Δ vs WT in SMG1i.
(TSV)

S12 Table Log2 fold changes in RNA-seq (mRNA) and ribosome footprints (RPF), as well as ribosome occupancy (TE), and corresponding p values (pvalue.adjust) for all transcripts as computed by Xtail. For GIGYF2Δ in SMG1i vs WT in DMSO.
(TSV)

S13 Table Log2 fold changes in RNA-seq (mRNA) and ribosome footprints (RPF), as well as ribosome occupancy (TE), and corresponding p values (pvalue.adjust) for all transcripts as computed by Xtail. For WT in SMG1i vs WT in DMSO.
(TSV)

S14 Table GIGYF2 immunoprecipitation and mass spectrometry with RNase or spliceostatin A treatment. Immunoaffinity purification/mass spectrometry analyses of StrepII-GIGYF2 from untreated, RNase A and SSA treated HEK293T cells. Label free quantification (LFQ) intensities along with the average fold changes and statistical significance are reported. Searched with Andromeda and analyzed with Maxquant (1.6.7.0) and Perseus (1.6.7.0).
(XLSX)

S15 Table GIGYF2 immunoprecipitation and mass spectrometry with SMG1i treatment. Same as S14 Table, for SMG1i treated HEK293T cells. No p-values were computed for this single-replicate experiment.
(XLSX)

S16 Table Description of oligos used in this study. (TXT)

S17 Table Description of plasmids used in this study. (TSV)
