# Supplementary figures and images for: Translational repression of NMD targets by GIGYF2 and EIF4E2
Source: PLoS Genet. 2021 Oct 19;17(10):e1009813. doi: 10.1371/journal.pgen.1009813 (PMC8555832; doi:10.1371/journal.pgen.1009813)

**A**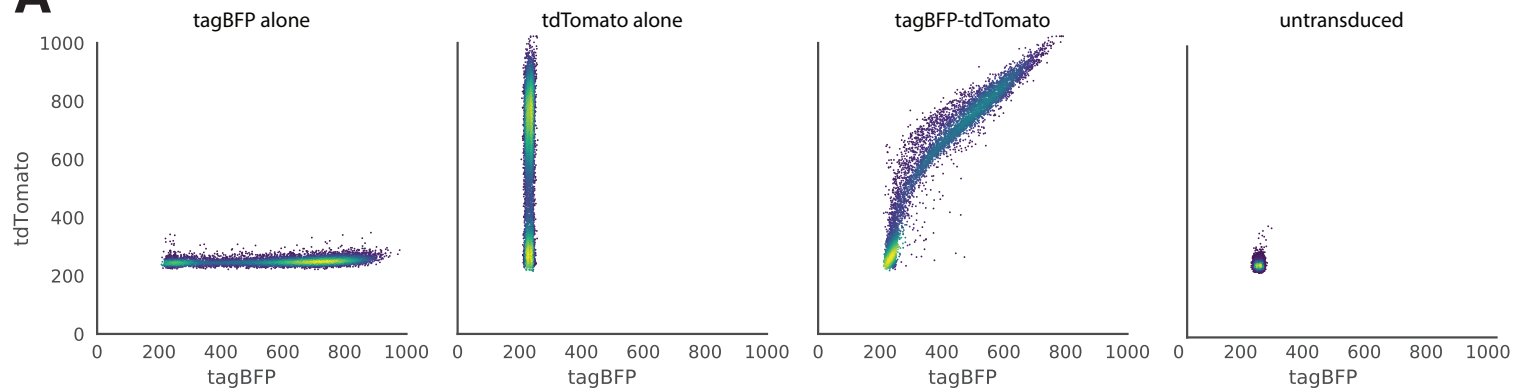**B**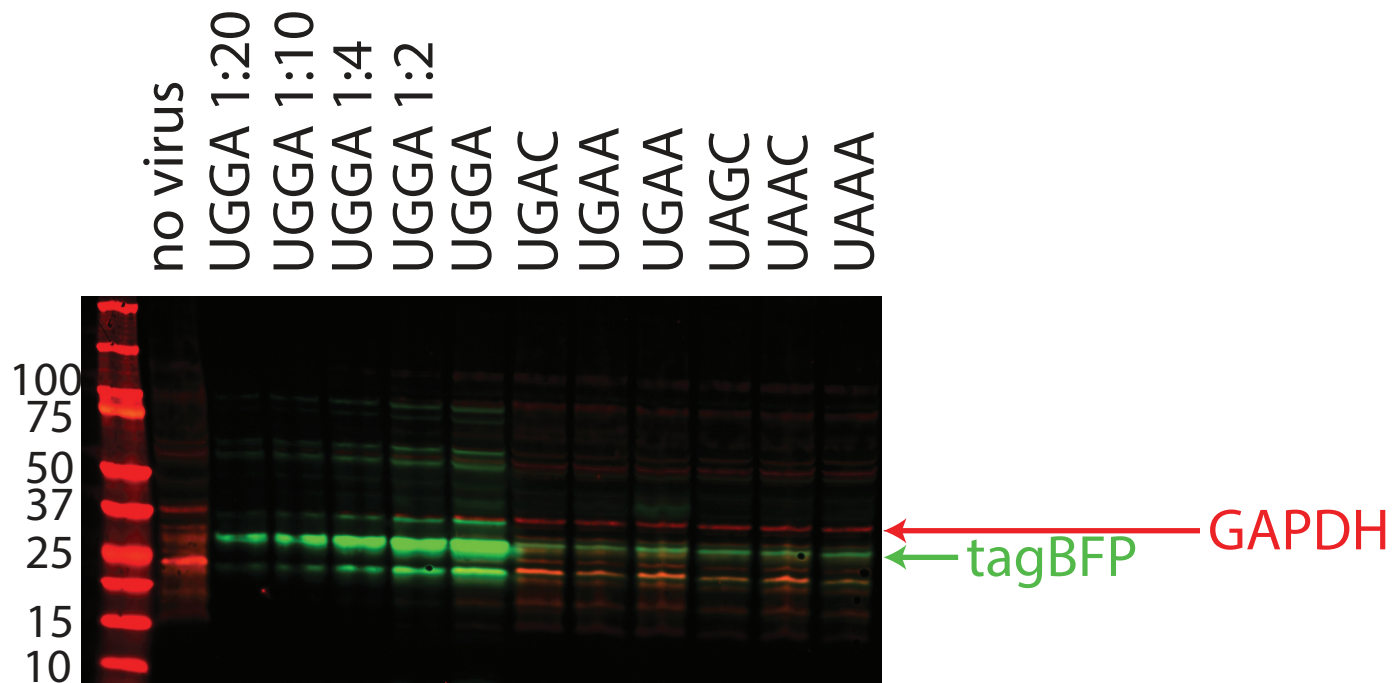

Supplement: S1 Fig — A) Flow cytometry of HEK293FT cells lentivirally transduced with indicated plasmids. tagBFP-tdTomato is the full reporter in Fig 1A with a UGG tryptophan codon at the PTC postion (Same as S1B Fig). B) Western blot of lysates from HEK293FT cells transduced with lentivirus containing reporters with the indicated stop codons, or UGG codon encoding tryptophan. Serial dilutions of UGG codon are also included. (PDF) [file pgen.1009813.s001.pdf]

**A**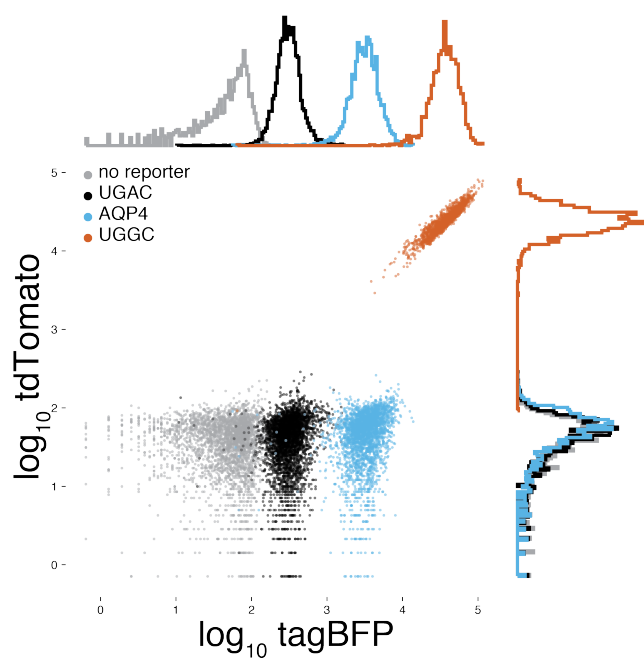**B**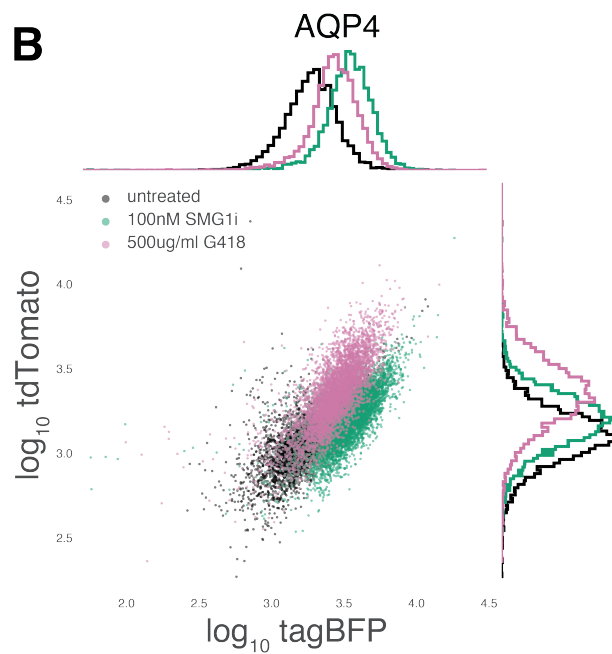**C**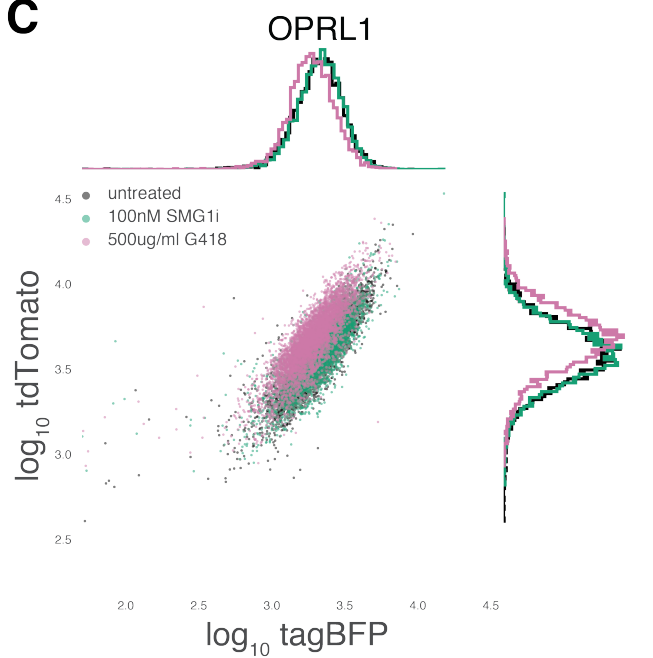**D**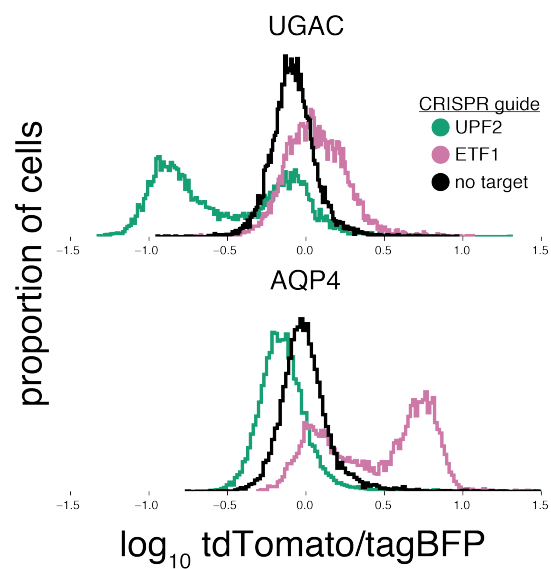

Supplement: S2 Fig — A) Flow cytometry of monoclonal K562 cells expressing reporter with different stop codon contexts (or a UGG tryptophan codon). B) Flow cytometry of monoclonal K562 cells expressing reporter with AQP4 stop context PTC, treated with SMG1i or G418 for 24 hours. C) Same as panel B, for OPRL1 PTC reporter. D) Histograms of tdTomato/tagBFP signal for flow cytometry data from Fig 1E and 1F. (PDF) [file pgen.1009813.s002.pdf]

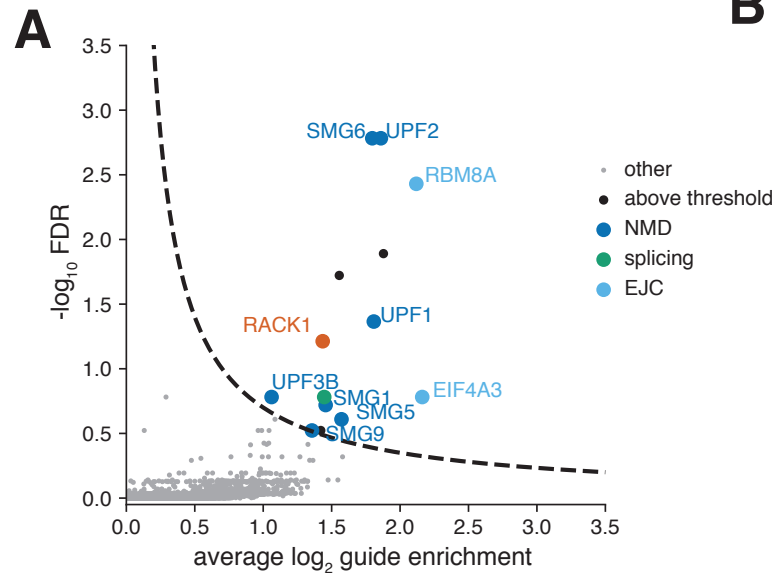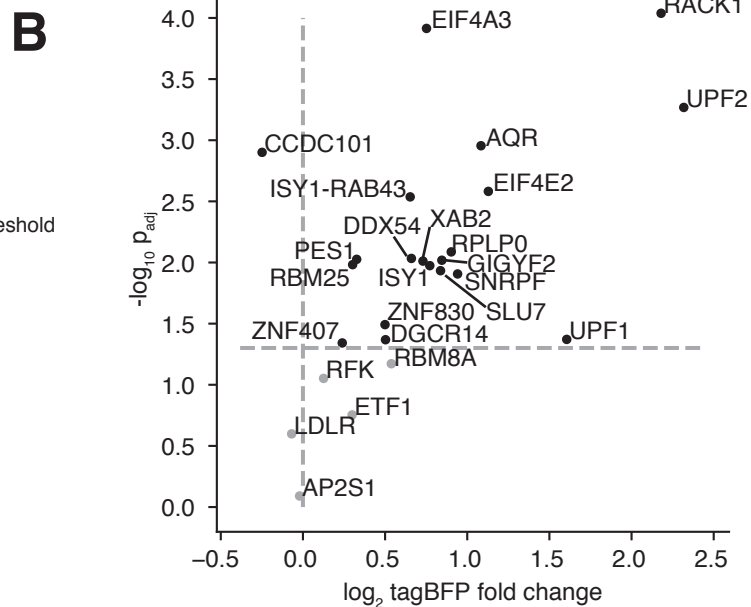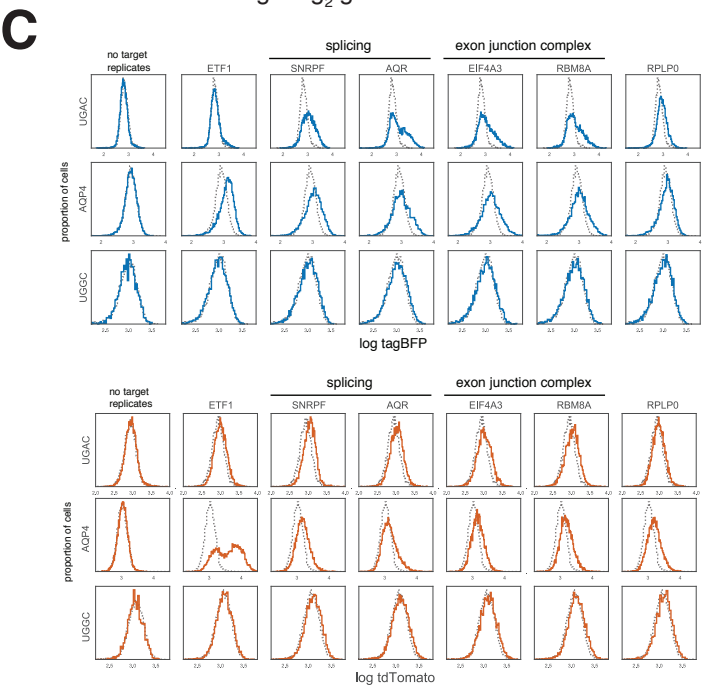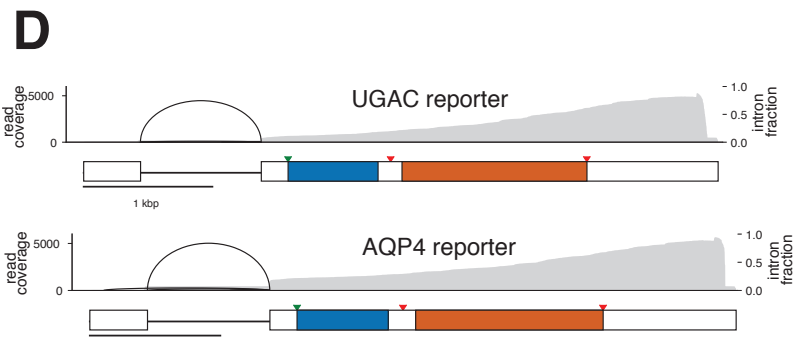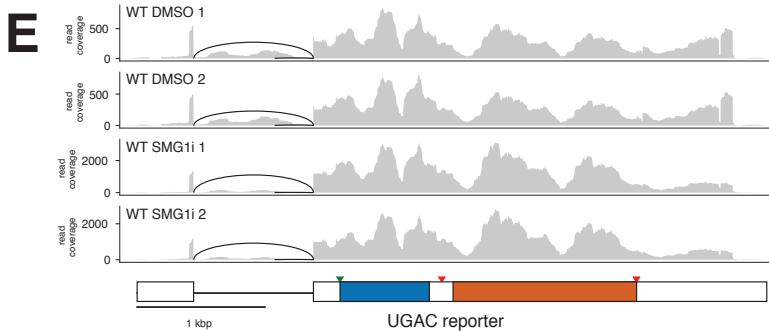

Supplement: S3 Fig — A) Volcano plot of fold enrichment and false discovery rate for CRISPR guides sequenced from cells with decreased tdTomato/tagBFP ratio compared to the input population for the AQP4 reporter. B) Volcano plot of fold changes and adjusted t-test p values for validation of select hits with the UGAC reporter. Each point is the relative mean tagBFP fluorescence (with average of nontargeting set to 1) of 2 or 3 separate lentiviral infections each with 2 separate guides (except for UPF1 and ETF1 controls, for which a single guide was used). Each t-test compares these 4 or 6 values against the 6 values for the nontargeting guides (2 separate guides in 3 separate infections). See S3 Table for individual values. The mean values include all cells in the sample, and therefore are affected by variations in transduction efficiency, CRISPR efficiency and knockout viability in addition to the magnitude of the effect on the reporter from the mutation. C) Histograms of tagBFP (blue line) or tdTomato (vermilion line) signal from flow cytometry of reporter lines lentivirally transduced with nontargeting guide (gray dotted line) or targeting guide (solid line), for reporter lines containing UGAC stop codon, AQP4 stop context, or no PTC (UGGC). SNRPF and AQR are splicing factors, RBM8A and EIF4A3 are core components of the EJC, and RPLP0 is a protein of the large ribosomal subunit. These samples were measured at 4 days post infection. D) Coverage of sequencing reads mapping to reporter from nanopore direct RNA sequencing of HEK293FT cells transfected with UGAC or AQP4 reporter plasmid. Fraction of reads at a position with a GU-AG splice junction are indicated as arcs, for introns with at least 2 supporting reads and making up at least 1% of reads spanning the intron ends. Reporter diagram indicates tagBFP and tdTomato coding regions as well as start and stop codons. E) Coverage of uniquely mapping paired-end RNA-seq reads mapping to integrated reporter in K562 reporter line treated with [file pgen.1009813.s003.pdf]

A

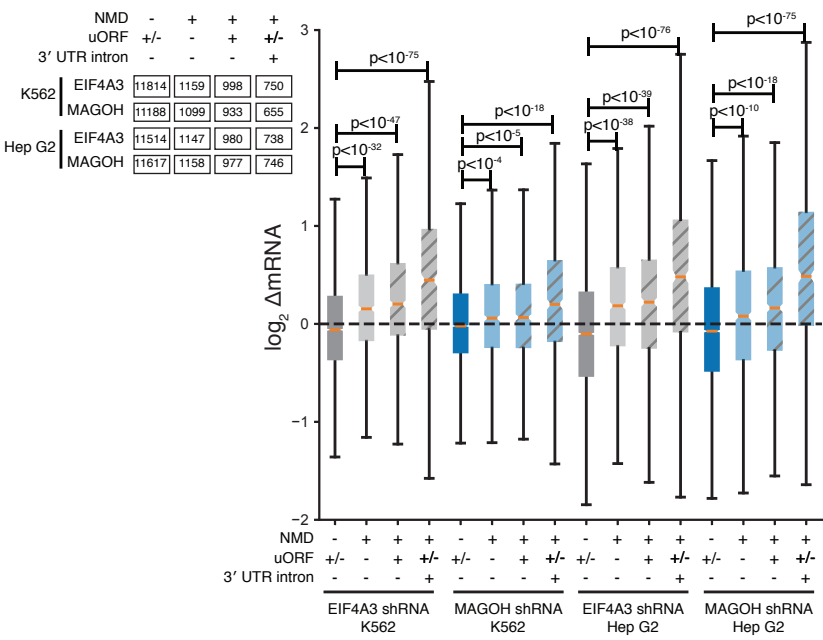

B

Hep G2 knockdown (ENCODE)

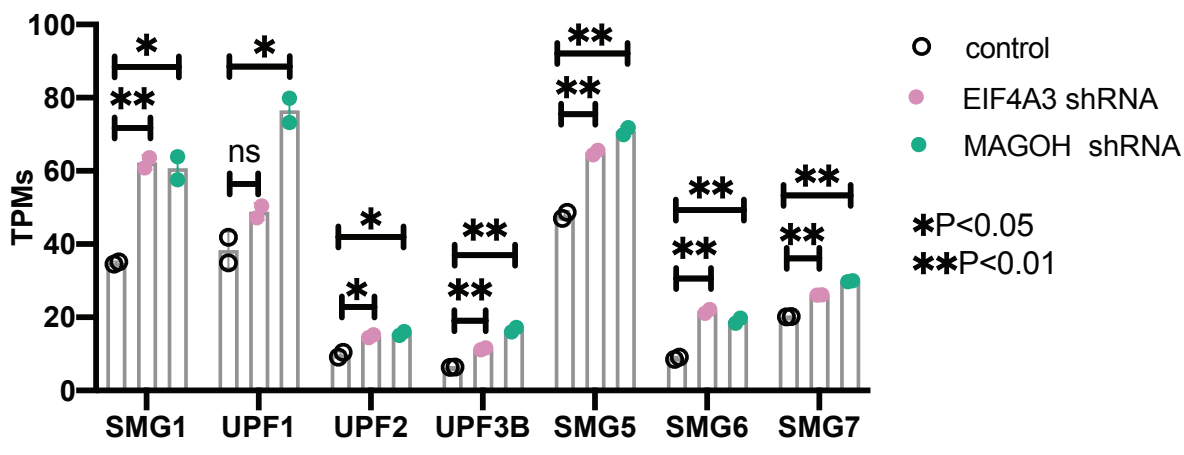

C

K562 knockdown (ENCODE)

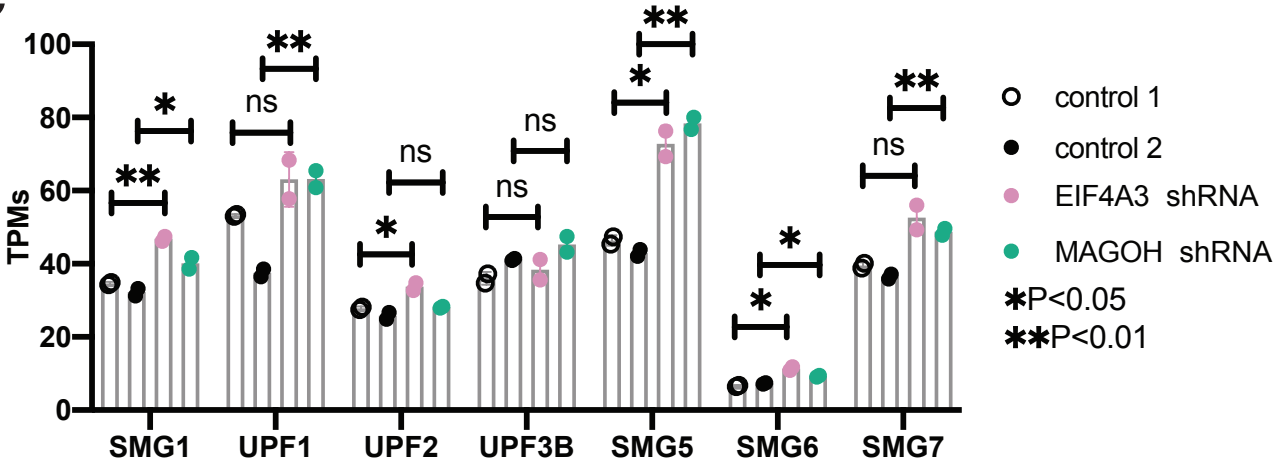

Supplement: S4 Fig — A) Box plot of fold changes in mRNA levels for EIF4A3 or MAGOH knockdowns in HepG2 or K562 cells from ENCODE. Transcript sets are divided into all transcripts, empirically determined NMD targets with or without uORFs, and the subset of these NMD targets with 3′ UTR introns. Transcript counts for each set are indicated at left, and p values from Mann-Whitney U-test are indicated. +/- indicates that transcripts both with and without uORFs are included in the transcript set. B) Transcripts per million (TPM) from ENCODE RNA-seq data in Hep G2 cells for a subset of transcripts encoding NMD factors. T-test p values are indicated as asterisks. C) Same as panel B, for K562 cells. Note that in this panel, control 1 is the matched control for eIF4A3 knockdown, while control 2 is the matched control for MAGOH knockdown. (PDF) [file pgen.1009813.s004.pdf]

**A**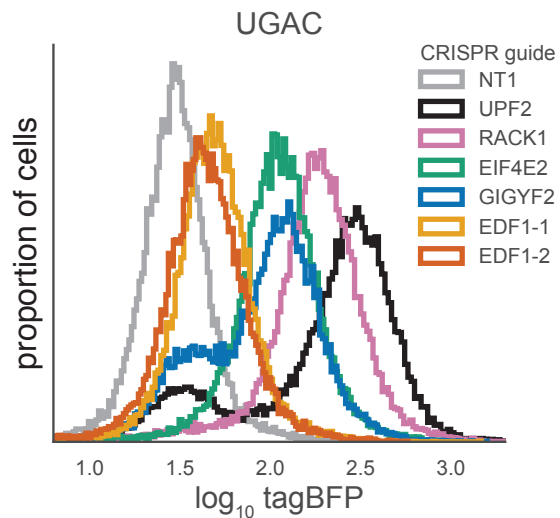**B**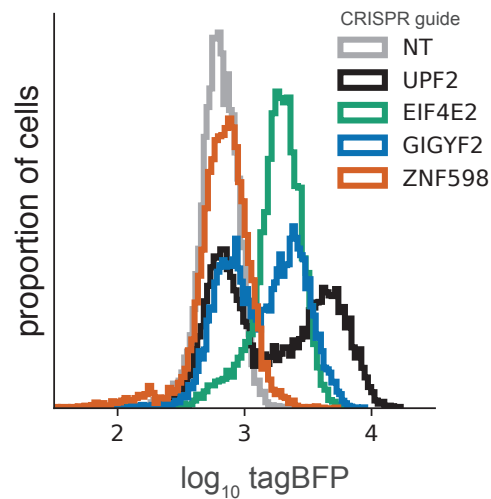**C**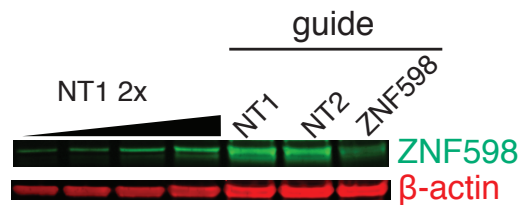**D**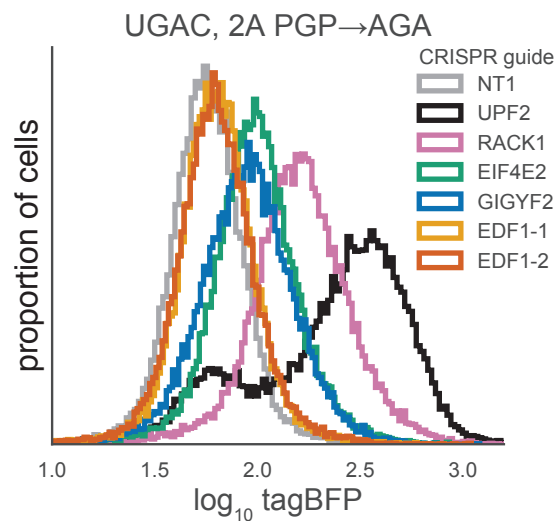

Supplement: S5 Fig — A) As in Fig 2C, with guides against EDF1 added. Note that all samples except EDF1 are the same ones as in Fig 2C, as these electroporations were performed simultaneously. B) tagBFP signal from flow cytometry of reporter lines transduced with nontargeting guide (NT1) or guides against indicated genes for reporter line containing UGAC stop codon. These samples were measured at 6 days post infection. C) Western blot of cell lines expressing nontargeting guides or a guide against ZNF598 from the same infections used for S3B Fig. Lysates were collected on the same day that the flow cytometry was performed. D) TagBFP intensities for independently generated reporter cell line with the 2A peptide upstream of the PTC disabled by mutating the terminal PGP residues to AGA. Electroporation and flow cytometry were performed in parallel with samples in panel A. Note that differences in signal intensity between the lines are likely due to differences in integration site and number, as well as stochastic variation in the monoclonal selection process. (PDF) [file pgen.1009813.s005.pdf]

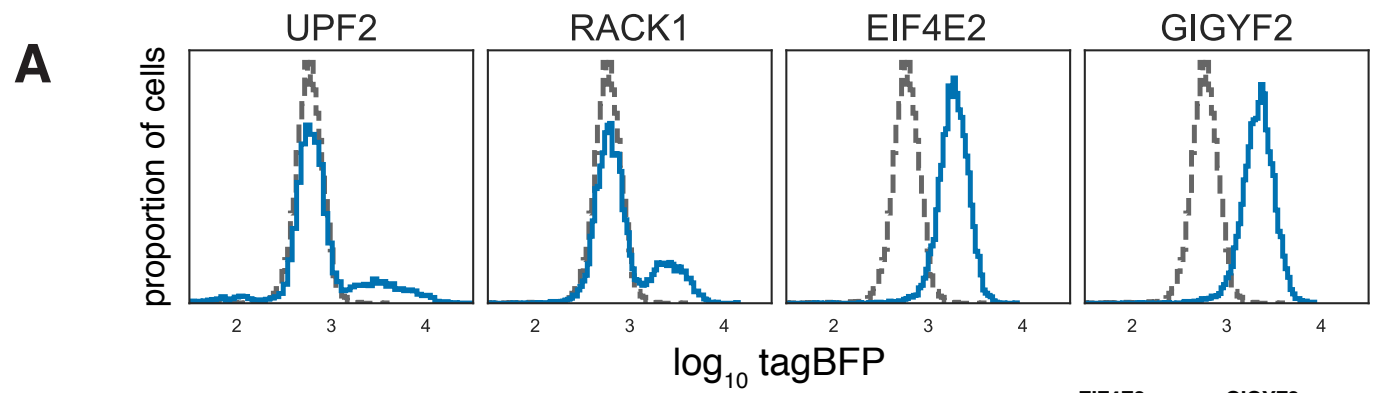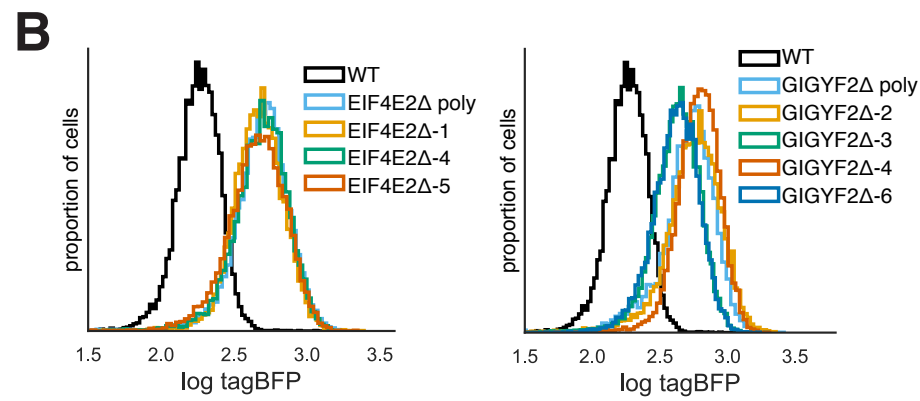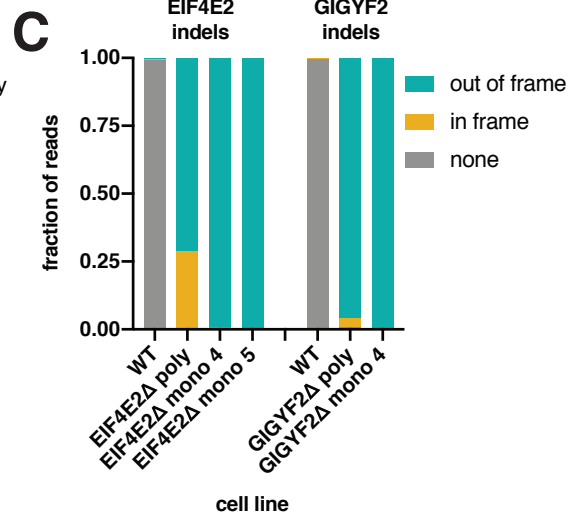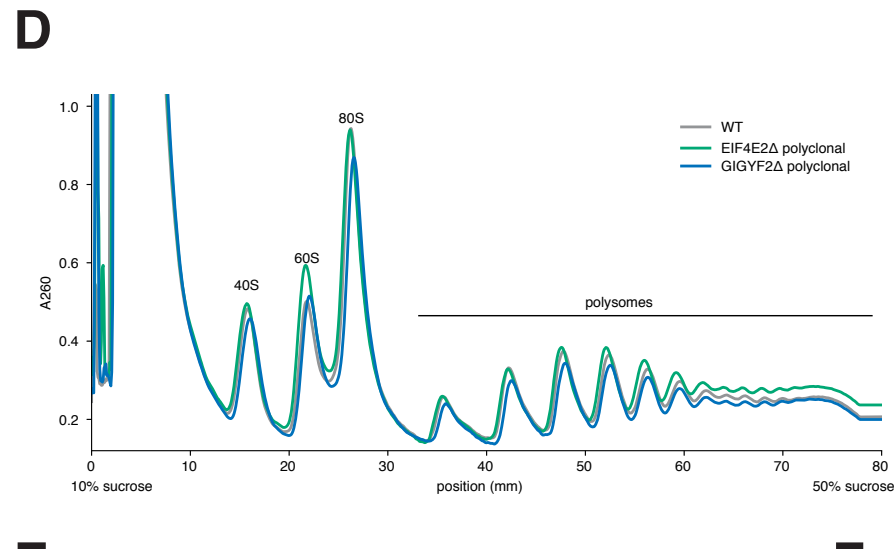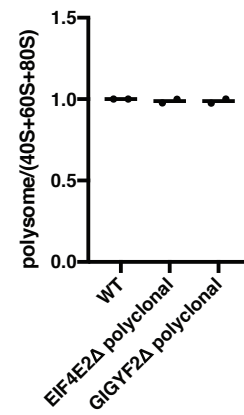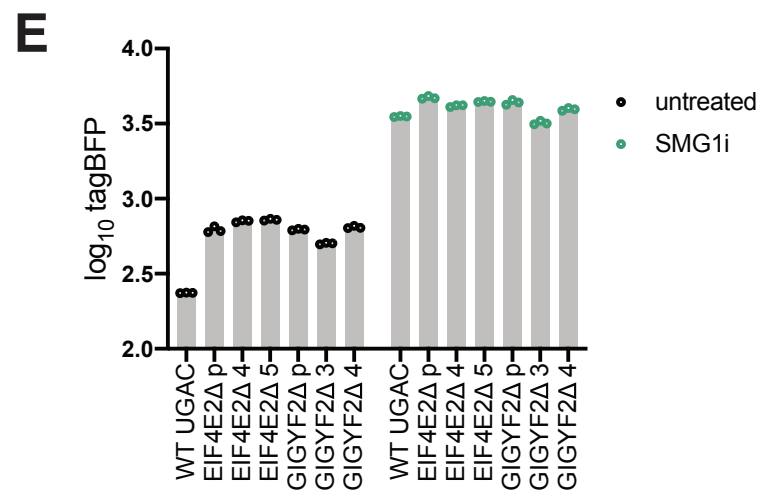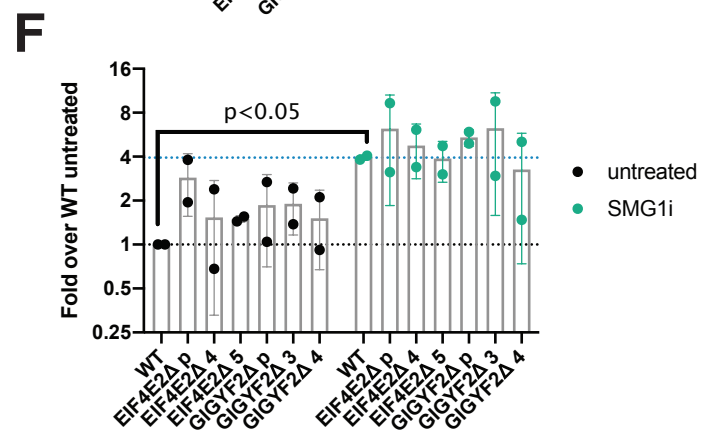

Supplement: S6 Fig — A) Histograms of tagBFP signal from flow cytometry of UGAC reporter lines infected with nontargeting guide (dotted gray line) or targeting guide (solid blue line) 42 days after infection, and 12 days after sorting for 5000 cells with increased tagBFP signal. The polyclonal populations expressing GIGYF2 and EIF4E2 guides were used for further experiments, and for production of monoclonal lines. B) tagBFP signal from WT UGAC reporter line, and GIGYF2Δ or EIF4E2Δ polyclonal or monoclonal lines. C) Allele frequencies in monoclonal or polyclonal GIGYF2Δ and EIF4E2Δ lines, as assessed by high-throughput sequencing of the CRISPR target site. D) (left)UV absorbance trace from 10–50% sucrose density gradient fractionation of WT and GIGYF2Δ or EIF4E2Δ polyclonal lines. (right) Quantification of ratio of polysome area to 40S+60S+80S area (normalized to WT sample processed on same day) from 2 independent replicates. E) Median tagBFP intensities for indicated WT or EIF4E2Δ/GIGYF2Δ cell lines, either untreated (black circles), or treated with SMG1i for 24 hours (green circles). Biological replicate treatments were performed on 3 separate days. F) Quantification of automated western blotting of tagBFP relative to β-actin, for indicated WT or EIF4E2Δ/GIGYF2Δ cell lines, either untreated (black circles), or treated with 300nM SMG1i for 24 hours (green circles). Biological replicates were collected and blotted on 2 separate days and normalized to the untreated WT sample within each day. These samples are distinct from the ones used for flow cytometry. Dotted lines show the means for the untreated and SMG1i treated WT samples. Error bars show standard deviation. P value computed by two-tailed ratio paired t-test. All unmarked comparisons had p>0.05. (PDF) [file pgen.1009813.s006.pdf]

**A**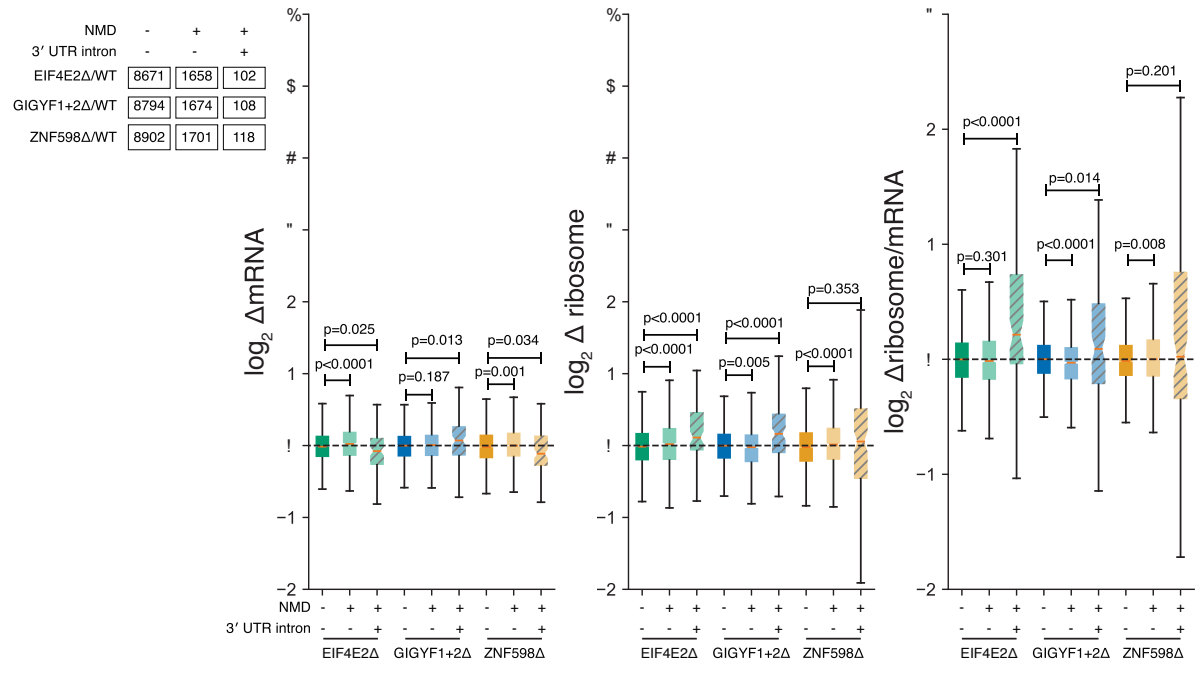**B**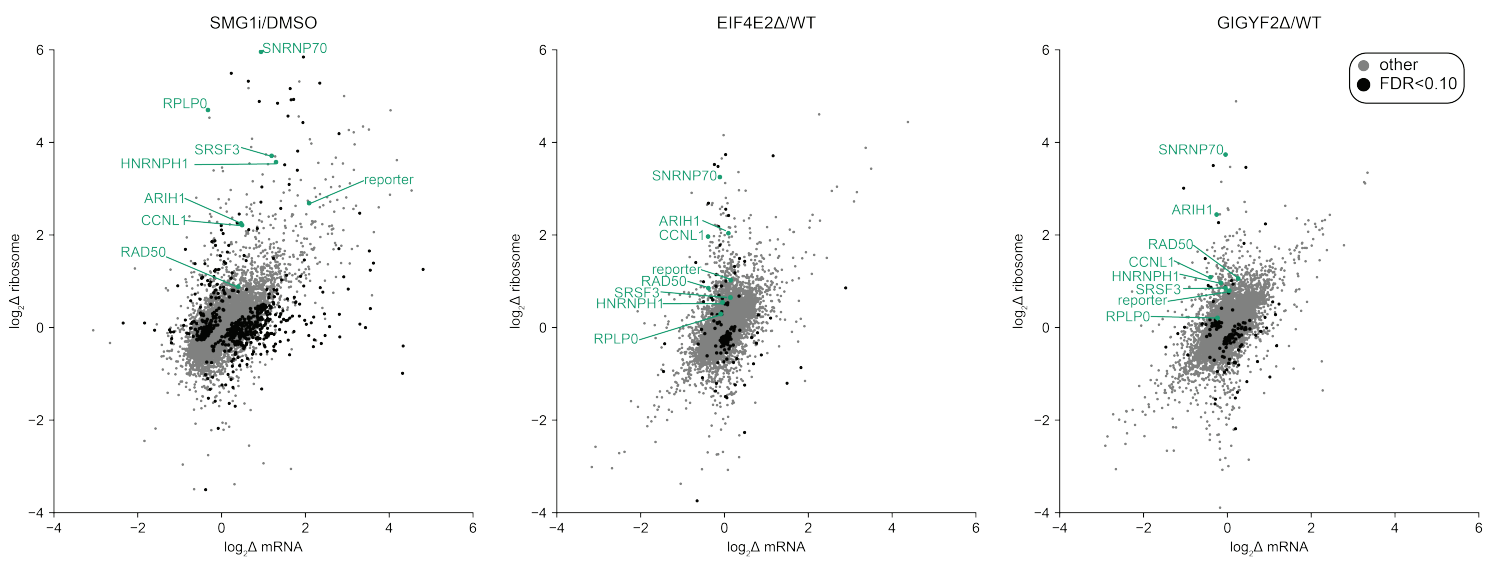**C**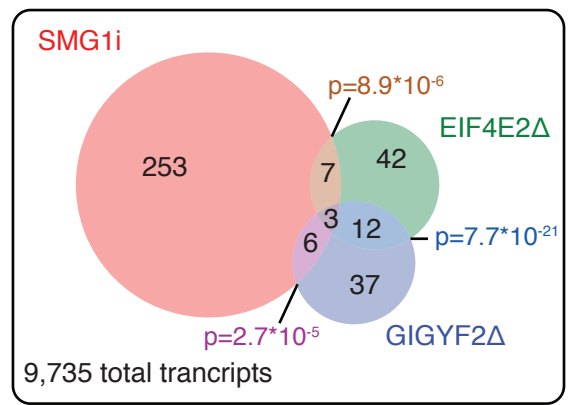**D**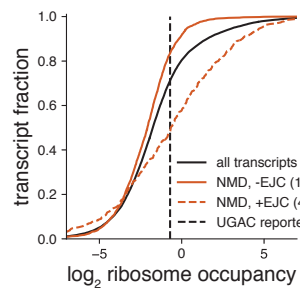**E**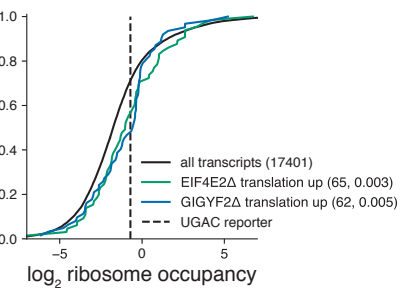

Supplement: S7 Fig — A) Box plot of fold changes in mRNA levels, ribosome footprints and ribosome occupancy (ratio of ribosome/mRNA) in EIF4E2Δ (green), GIGYF1Δ/GIGYF2Δ (blue), or ZNF598Δ HEK293T cells, relative to WT cells, for all transcripts (dark bars), empirically determined NMD targets (faded bars), and the subset of these targets with 3′ UTR introns (faded hatched bars). Transcript counts for each set are indicated at left, and p values from Mann-Whitney U-test are indicated. Raw data are from [57]. B) Comparison of fold changes in mRNA levels with fold changes in ribosome footprints for all quantified transcripts. Transcripts passing false discovery rate cutoff of 0.1 for changes in ribosome occupancy are shaded in black. Transcripts mentioned in the text are labelled in green. C) Venn diagram of transcripts passing cutoff from Fig 5A. P values for overlaps were computed by hypergeometric test, using a background set of 9,735 transcripts with computable fold changes in all datasets. D) Cumulative distribution of ribosome densities in EIF4E2Δ cells for NMD targets without (-EJC, solid orange line) or with (+EJC, dashed orange line) 3′ UTR introns. The number of transcripts in each set, and p value for Mann-Whitney U test against all transcripts (black line) is indicated in parentheses. The vertical black dotted line indicates the ribosome occupancy of the UGAC reporter. E) Same as panel D, but for the transcripts with increased ribosome occupancy in EIF4E2Δ (green line) or GIGYF2Δ (blue line). (PDF) [file pgen.1009813.s007.pdf]

**A**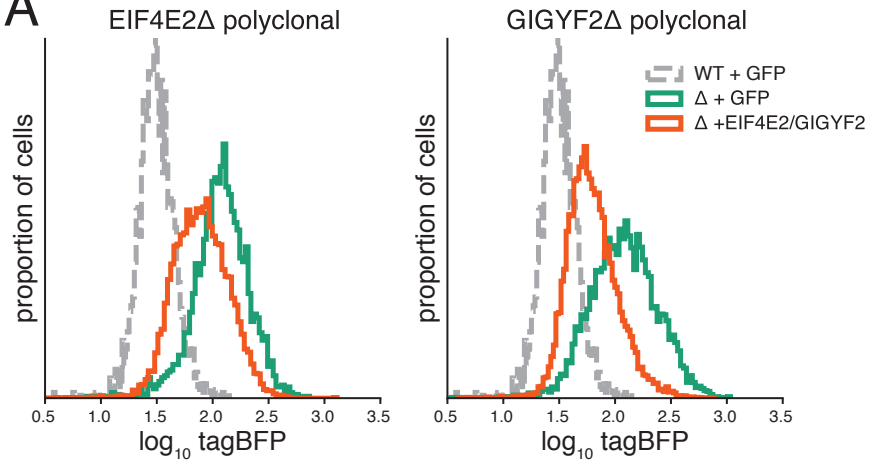**B**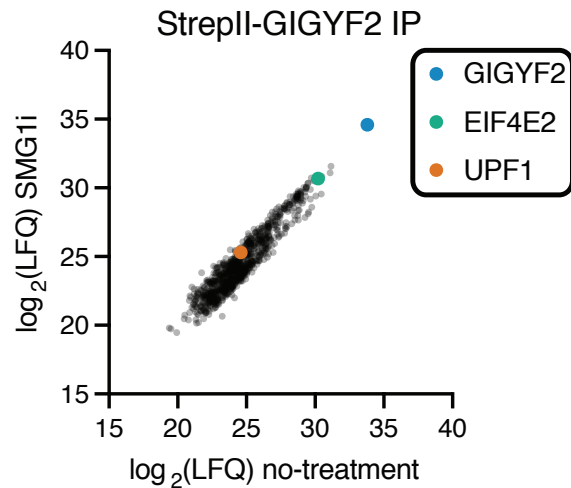

Supplement: S8 Fig — A) Flow cytometry analysis of rescue of EIF4E2Δ or GIGYF2Δ UGAC reporter polyclonal lines by electroporation of the GIGYF2:EIF4E2 coexpression plasmid used in Fig 6A–6E, with CRISPR guide sites mutated. B) Mass spectrometry analyses of StrepII-GIGYF2 immunoprecipitates with or without SMG1i treatment. Scatter plot showing log2(LFQ) intensity of proteins identified under SMG1i (y-axis) and untreated (x-axis) regimes. Values are from a single replicate of each sample. (PDF) [file pgen.1009813.s008.pdf]
